# Planned mode of birth after previous cesarean section: A structured review of the evidence on the associated outcomes for women and their children in high-income setting

**DOI:** 10.3389/fmed.2022.920647

**Published:** 2022-09-06

**Authors:** Kathryn E. Fitzpatrick, Maria A. Quigley, Jennifer J. Kurinczuk

**Affiliations:** National Perinatal Epidemiology Unit, Nuffield Department of Population Health, University of Oxford, Oxford, United Kingdom

**Keywords:** elective repeat cesarean section (ERCS), vaginal birth after previous cesarean delivery (VBAC), trial of labor after a previous cesarean delivery, maternal outcomes, child outcomes, perinatal outcomes, vaginal birth after previous cesarean section, planned mode of birth after previous cesarean section

## Abstract

In many high-income settings policy consensus supports giving pregnant women who have had a previous cesarean section a choice between planning an elective repeat cesarean section (ERCS) or planning a vaginal birth after previous cesarean (VBAC), provided they have no contraindications to VBAC. To help women make an informed decision regarding this choice, clinical guidelines advise women should be counseled on the associated risks and benefits. The most recent and comprehensive review of the associated risks and benefits of planned VBAC compared to ERCS in high-income settings was published in 2010 by the US Agency for Healthcare Research and Quality (AHRQ). This paper describes a structured review of the evidence in high-income settings that has been published since the AHRQ review and the literature in high-income settings that has been published since 1980 on outcomes not included in the AHRQ review. Three databases (MEDLINE, EMBASE, and PsycINFO) were searched for relevant studies meeting pre-specified eligible criteria, supplemented by searching of reference lists. Forty-seven studies were identified as meeting the eligibility criteria and included in the structured review. The review suggests that while planned VBAC compared to ERCS is associated with an increased risk of various serious birth-related complications for both the mother and her baby, the absolute risk of these complications is small for either birth approach. The review also found some evidence that planned VBAC compared to ERCS is associated with benefits such as a shorter length of hospital stay and a higher likelihood of breastfeeding. The limited evidence available also suggests that planned mode of birth after previous cesarean section is not associated with the child’s subsequent risk of experiencing adverse neurodevelopmental or health problems in childhood. This information can be used to manage and counsel women with previous cesarean section about their subsequent birth choices. Collectively, the evidence supports existing consensus that there are risks and benefits associated with both planned VBAC and ERCS, and therefore women without contraindications to VBAC should be given an informed choice about planned mode of birth after previous cesarean section. However, further studies into the longer-term effects of planned mode of birth after previous cesarean section are needed along with more research to address the other key limitations and gaps that have been highlighted with the existing evidence.

## Introduction

Cesarean sections are now one of the most common surgical procedures performed, with many parts of the world having seen a sharp rise in their cesarean section rates in recent years (1–4). The rise in cesarean section rates has led to increasing numbers of pregnant women with a history of previous cesarean section. The optimal birth approach in this situation, whether to plan another cesarean known as an elective repeat cesarean section (ERCS) or plan a vaginal birth, known as a planned vaginal birth after previous cesarean (VBAC), has long been a debated issue (5, 6). Current clinical guidelines in many high-income countries support giving women a choice between planned VBAC or ERCS, provided they do not have contraindications to VBAC (7–11). To help women make an informed decision regarding this choice, clinical guidelines advise women should be counseled on the associated risks and benefits. A number of systematic reviews of the evidence concerning the risks and benefits of planned VBAC compared to ERCS have been conducted (12–19), the most recent and comprehensive of which was published in 2010 by the US Agency for Healthcare Research and Quality (AHRQ) (20). The AHRQ review summarized the literature between 1980 and September 2009 relating to women with a singleton pregnancy in developed countries who had undergone one or more previous cesarean sections, with 41 studies on maternal outcomes and 11 studies on neonatal outcomes identified. The AHRQ review highlighted several significant limitations with the evidence. These included a lack of comparability between the comparison groups, with it often being unclear whether women included in the ERCS group were truly eligible to plan a VBAC, and inferring intended mode of birth from actual mode of birth. The AHRQ review also identified several specific areas particularly lacking robust evidence or any evidence at all, including the effect of planned VBAC compared to ERCS on the following outcomes: maternal hemorrhage, maternal infection, maternal surgical injury, breastfeeding initiation or continuation, women’s risk of pelvic floor dysfunction/perineal trauma, neonatal respiratory intervention/morbidity, hypoxic-ischemic encephalopathy/asphyxia, neonatal sepsis, birth trauma, admission to a neonatal intensive care unit (NICU), and the child’s neurological development. Also of note is that much of the literature identified by the AHRQ review consisted of non-population-based cohort studies conducted in a single or small number of mainly tertiary or academic medical institutions. Such studies tend to be prone to several limitations such as limited generalizability and lack of statistical power. While the AHRQ review represents the most recent and comprehensive review of outcomes associated with planned mode of birth after previous cesarean section, as well as being published over 12 years ago now, the review did not consider certain important outcomes that are thought to have a plausible association with mode of birth including women’s mental health and health problems in childhood (21–28). This paper describes a structured review of the literature in high-income settings that has been published since the AHRQ review as well as the literature in high-income settings that has been published since 1980 on outcomes not included in the AHRQ review. In doing so, this paper aimed to provide a comprehensive up-to-date overview of the evidence on the short and longer-term outcomes for women and their children following planned VBAC compared to ERCS in high-income settings to help facilitate informed decision making about this choice. We also aimed to highlight remaining limitations and gaps with the existing evidence.

## Methods

### Study design and eligibility criteria

A structured review of the literature was conducted, with studies eligible for inclusion if they included women with one or more prior cesarean sections and presented original data sufficient to compare outcomes for women and/or their children associated with planned VBAC vs. ERCS.

Studies were excluded using similar criteria to those used in the AHRQ systematic review (20). This included if they: had 10 or fewer participants; focused on women without a prior cesarean birth, nulliparous women, breech birth or women with particular conditions such gestational diabetes, human immunodeficiency virus, and preeclampsia; exclusively focused on preterm (< 37 weeks’ gestation) birth, low birth weight, small for gestational age, multiple births, abortions, or antepartum stillbirths; were non-English language papers; were not conducted in a high income setting as defined by the World Bank in 2022; were editorials or letters; were available exclusively in abstract form; or were studies of animals or cadavers. When examining the outcomes perinatal or neonatal mortality, studies which did not exclude infants with known congenital or lethal anomalies from the analysis were also excluded as was done in the AHRQ systematic review (20).

### Outcomes

The following maternal outcomes were considered: mortality; uterine rupture; hysterectomy for complications resulting from birth; hemorrhage; blood transfusion; infection; surgical injury; length of hospital stay; breastfeeding; pelvic floor dysfunction/perineal trauma; mental health.

Baby/child outcomes considered included: perinatal and neonatal mortality; neonatal respiratory intervention/morbidity (including ventilation, intubation, need for oxygen, and transient tachypnea of the newborn); hypoxic-ischemic encephalopathy/asphyxia; neonatal sepsis/infection; birth trauma; admission to a neonatal intensive care unit (NICU); low Apgar score (<7 or <4 at 5 min after birth); neurological development; health problems in childhood (including obesity, cancer, infections, type-1 diabetes, asthma, and inflammatory bowel disease).

All of these outcomes are thought to have a plausible association with mode of birth (21–28) and are outcome measures that were considered to be important in relevant systematic reviews (18, 20, 29, 30). Representatives of various service user and voluntary groups in the perinatal field were also consulted and their views about what outcomes they considered to be important to women and their partners when deciding on mode of birth after previous cesarean section were considered when determining which outcome measures to include.

### Search strategy

MEDLINE, EMBASE, and PsycINFO were searched for relevant studies. The search strategy used in the AHRQ systematic review (20) was updated and used to search for papers published since that review. Additional search strategies focusing on the outcomes not described in the AHRQ review (women’s mental health and health problems in childhood) were also used to search for studies published from 1980 that included these outcomes (see Supplementary Material for search strategies). Searches were performed up to March 2022. The references of all included papers and relevant systematic reviews were also reviewed to identify additional articles which may have been missed by the search strategies.

### Data extraction and analysis

Having first removed duplicates, the titles and abstracts of articles identified by the search strategies were screened against the eligibility criteria. The full texts of potentially eligible articles were then reviewed against the eligibility criteria. Data were extracted into a standardized form for each of the included studies. Title and abstract and full-text screening of articles as well as data extraction were all performed by a single author (KF). Where a study did not report a relative measure of effect [e.g., a risk ratio (RR) or odds ratio (OR)], this was calculated using the *csi* function in Stata 13 SE where there was sufficient information provided to do so. In situations where there was a small number (<5) of outcome events in a category, the penalized maximum likelihood estimation proposed by Firth (Stata add-in command *firthlogit*) was used to try and overcome the potential issue of sparse-data bias that can occur in maximum likelihood estimation (31). As this was a structured review, meta-analysis which is frequently used in systematic reviews was not performed but forest plots were used to graphically display results along with the relevant AHRQ systematic review (20) results for comparison where available. Also, in contrast to what is typically done in a full systematic review, no formal evaluation of the risk of reporting bias or assessment/rating of the quality of the included studies was performed.

## Results

Having removed duplicates, a total of 5,840 articles were identified, 5,597 of which were excluded after title and abstract screening (Supplementary Figure 1). After reviewing the full text of the remaining articles, 47 were identified as meeting the eligibility criteria and were included in the structured review.

### Characteristics of studies included in structured review

Supplementary Table 1 outlines the characteristics of the 47 studies included in the structured review. There were two small randomized controlled trials among the included studies (32, 33), one of which was nested within a patient preference prospective cohort study (33). Of the other included studies, 44 used a cohort design (34–77) and one was a case-control study (78). Twenty-eight were population-based studies (34, 36, 38, 41–43, 45, 50, 51, 53–56, 58–60, 63–65, 67, 69–71, 73, 75–78), while 13 recruited subjects from a single (32, 37, 39, 44, 46–48, 57, 61, 62, 68, 72, 74) and six from multiple centers (33, 35, 40, 49, 52, 66). Eighteen of the studies were conducted in mainland Europe (34, 37, 39, 41, 43, 46, 48, 49, 51, 56–58, 60, 62, 63, 66–68), 10 in the United States (35, 40, 42, 45, 52, 55, 59, 70, 71, 77), six in the United Kingdom (53, 54, 65, 75, 76, 78), four in Australia (33, 36, 38, 69), three in Israel (44, 47, 72), two in Canada (50, 64), two in Japan (73, 74), one in Hong Kong (32) and one in Taiwan (61). The studies involved a median of 27,007 subjects (range 50–1,833,407) and the recruitment period ranged from 1982 to 2019. Just over half of the studies were restricted to women or the children of women with one prior cesarean section (*n* = 25); the majority excluded multiple births (*n* = 36); and more than half were confined to women, or the children of women delivered at term (*n* = 27). Studies varied greatly in the extent to which they attempted to exclude births to women with contraindications to VBAC. They also varied in the degree to which they adjusted for potential confounders and very few tested for effect modification between mode of birth and covariates. Most of the studies reported data on more than one outcome. Findings by outcome are described below.

#### Maternal outcomes

##### Mortality

Nine studies (33, 40, 46, 47, 50, 55, 66, 68, 73) (three population-based, eight of women who gave birth at term) involving a median of 7,755 women (range 412–685,137) were identified as reporting on maternal mortality in relation to planned VBAC and ERCS since the publication of the AHRQ review (20). Only four maternal deaths occurred, all in women who underwent an ERCS.

##### Uterine rupture

**FIGURE 1:**
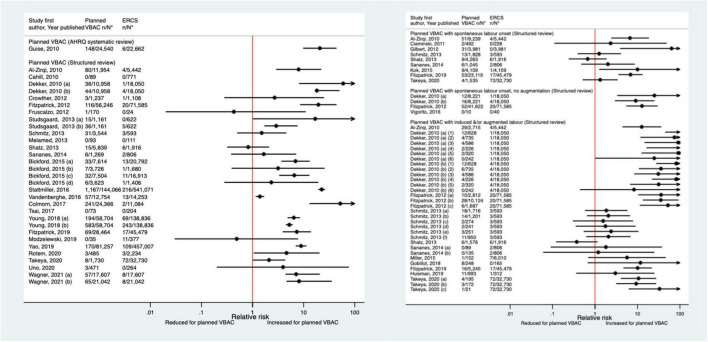
Risk of uterine rupture for planned VBAC vs. ERCS. *n, number in group with the outcome; *N*, Total number in group. Dekker et al. (36) (a) outcome: complete uterine rupture, (b) outcome: partial uterine rupture, (1) planned VBAC with spontaneous onset of labor, augmentation with oxytocin, (2) planned VBAC with induced labor onset, oxytocin only, (3) planned VBAC with induced labor onset, prostaglandins only, (4) planned VBAC with induced labor onset, oxytocin and prostaglandins, (5) planned VBAC with induced labor onset, no oxytocin or prostaglandins, (6) planned VBAC with induced labor onset, unspecified method; Studsgaard et al. (48) (a) Outcome: complete uterine rupture, (b) Outcome: incomplete uterine rupture; Bickford and Janssen (50) (a) risk in women with 1–2 prior CSs only, (b) risk in women with 1–2 prior CSs & ≥ 1 prior VB, (c) risk in women with 1 prior CS only, (d) risk in women with 1 prior CS and ≥ 1 prior VB; Young et al. (64) (a) outcome: uterine rupture not including dehiscence, (b) outcome: uterine rupture including dehiscence; Fitzpatrick et al. (78) (a) planned VBAC where labor induced with prostaglandin and oxytocin not used in labor, (b) planned VBAC where labored without prostaglandin induction but oxytocin used in labor, (c) planned VBAC where labor induced with prostaglandin and oxytocin used in labor; Schmitz et al. (46) (a) planned VBAC with induction or augmentation of labor, (b) planned VBAC with oxytocin only, (c) planned VBAC with prostaglandins only, (d) planned VBAC with prostaglandins and oxytocin, (e) planned VBAC where labor induced with oxytocin, (f) planned VBAC where labor augmented with oxytocin; Sananes et al. (49) (a) planned VBAC where labor induced with oxytocin and amniotomy, (b) planned VBAC where induced with Foley catheter; Takeya et al. (73) (a) planned VBAC with induction or augmentation of labor, (b) planned VBAC with induction or augmentation of labor with oxytocin, (c) planned VBAC with induction or augmentation of labor with any prostaglandins with or without oxytocin. AHRQ, Agency for Healthcare Research and quality; CS, cesarean section; ERCS, elective repeat cesarean section; VBAC, vaginal birth after previous cesarean; VB, vaginal birth.

Since the AHRQ review (20), 31 studies (33–37, 39, 40, 44, 46–52, 55–58, 61, 62, 64–66, 68, 70, 72–74, 77, 78) (13 population-based, 18 of women who gave birth at term) were identified as reporting on uterine rupture in relation to planned VBAC and ERCS. Uterine rupture was variably defined amongst these studies and the reported absolute risk of rupture varied from 0.00 to 4.76% for planned VBAC and 0.00–2.92% for ERCS. Sixteen of the studies (34, 36, 40, 48, 50, 51, 55, 56, 58, 64, 65, 70, 72, 73, 77, 78) (seven restricted to women with one prior cesarean) reported an increased risk of uterine rupture for planned VBAC compared to ERCS (relative effect ranging from 1.39 to 243.98), with the highest risks generally apparent when labor was induced and/or augmented. However, one of the studies reported that the risk was only significantly elevated amongst women without a prior vaginal birth (50). The remaining predominately small studies found no significant difference in the risk of this outcome for planned VBAC compared to ERCS (33, 37, 39, 46, 47, 49, 52, 62, 66, 68, 74) (eight restricted to women with one prior cesarean) or reported no cases at all of uterine rupture (35, 44, 57, 61) (two restricted to women with one prior cesarean) (Figure 1).

##### Hysterectomy

**FIGURE 2:**
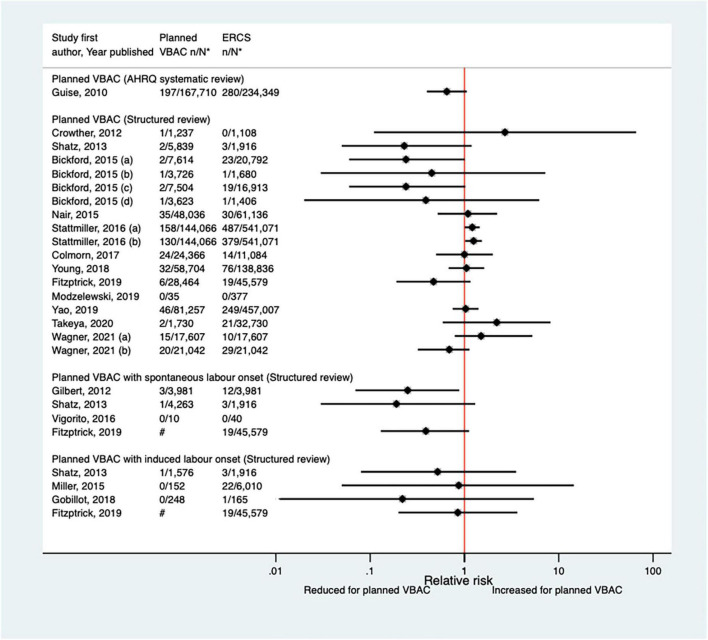
Risk of hysterectomy for planned VBAC vs. ERCS. *n, number in group with the outcome; *N*, Total number in group. Bickford and Janssen (50) (a) risk in women with 1–2 prior CSs only, (b) risk in women with 1–2 prior CSs and ≥ 1 prior VB, (c) risk in women with 1 prior CS only, (d) risk in women with 1 prior CS and ≥ 1 prior VB; Stattmiller et al. (55) (a) outcome: hysterectomy, (b) outcome: severe postpartum hemorrhage requiring hysterectomy. Wagner et al. (77) (a) Risk in women with no prior vaginal births. (b) Risk in women with ≥ 1 prior vaginal birth. ^#^Numbers have not been shown to protect against potential disclosure risks. AHRQ, Agency for Healthcare Research and quality; CS, cesarean section; ERCS, elective repeat cesarean section; VBAC, vaginal birth after previous cesarean; VB, vaginal birth.

The risk of hysterectomy associated with planned VBAC and ERCS was examined in sixteen studies (33, 40, 47, 50, 52, 53, 55, 57, 58, 62, 64, 65, 68, 70, 73, 77) (nine population-based, 12 of women who gave birth at term) since the AHRQ review (20). The absolute risk of hysterectomy in these studies varied from 0.00 to 0.12% for planned VBAC and 0.00–0.61% for ERCS. Of the 14 studies where cases of hysterectomy occurred, 12 (33, 47, 50, 52, 53, 58, 62, 64, 65, 70, 73, 77) found no significant difference in the risk of hysterectomy for planned VBAC compared to ERCS, while one study (40) reported a reduced risk of hysterectomy for planned VBAC (with spontaneous labor onset), adjusted odds ratio (aOR) 0.25, 95% CI 0.07–0.88, and one (55) (the largest study) reported a higher risk for planned VBAC, aOR ∼1.2 (Figure 2).

##### Hemorrhage

**FIGURE 3:**
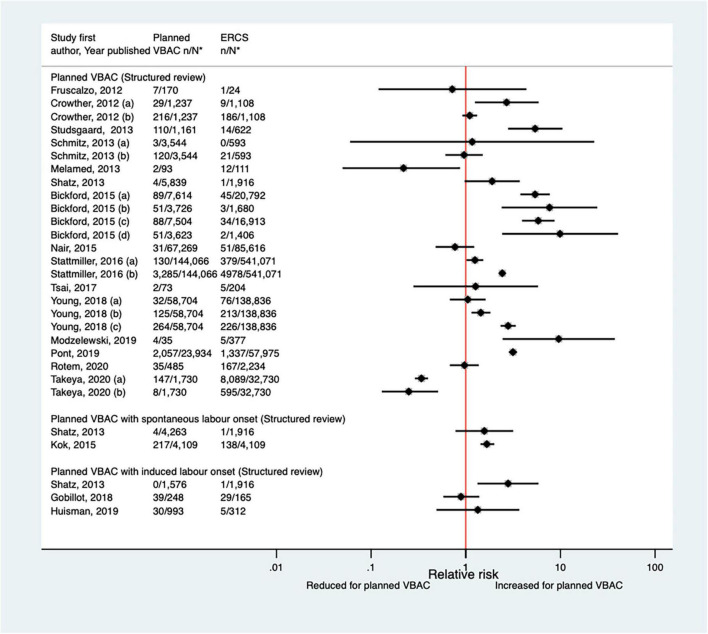
Risk of hemorrhage for planned VBAC vs. ERCS. *n, number in group with the outcome; *N*, Total number in group. Crowther et al. (33) (a) outcome: blood loss ≥ 1,500 mL and/or requiring blood transfusion, (b) outcome: blood loss > 500 mL; Schmitz et al. (46) (a) outcome: surgery (compression sutures, artery ligation, and hysterectomy) for postpartum hemorrhage, (b) outcome: prostaglandins for postpartum hemorrhage; Stattmiller et al. (55) (a) risk in women with 1–2 prior CSs only, (b) risk in women with 1–2 prior CSs and ≥ 1 prior VB, (c) risk in women with 1 prior CS only, (d) risk in women with 1 prior CS and ≥ 1 prior VB; Stattmiller et al. (55) (a) outcome: severe postpartum hemorrhage requiring hysterectomy, (b) outcome: severe postpartum hemorrhage; Young et al. (64) (a) outcome: postpartum hemorrhage requiring hysterectomy, (b) outcome: postpartum hemorrhage requiring procedures to control bleeding, (c) outcome: postpartum hemorrhage requiring blood transfusion; Takeya et al. (73) (a) outcome: hemorrhage, defined as ≥ 1,000 mL, (b) outcome: hemorrhage, defined as ≥ 2,000 mL. CS, cesarean section; ERCS, elective repeat cesarean section; VBAC, vaginal birth after previous cesarean; VB, vaginal birth.

Eighteen studies (33, 39, 44, 46–48, 50, 51, 53, 55, 61, 62, 64, 66, 68, 69, 72, 73) (seven population-based, 14 of women who gave birth at term) have examined the occurrence of hemorrhage for planned VBAC and ERCS since the AHRQ review (20). These studies variably defined hemorrhage and reported absolute risks ranged from 0.05 to 17.46% for planned VBAC and 0.00–24.71% for ERCS. Nine of the studies (33, 47, 48, 50, 51, 55, 64, 68, 69) reported an increased risk of hemorrhage for planned VBAC compared to ERCS (relative effect ranging from 1.25 to 9.90). However, for one of these studies (33) the risk was only significantly higher for major hemorrhage (defined as blood loss ≥ 1,500 mL and/or requiring a blood transfusion) while for another of the studies (47) the risk was only significantly elevated for planned VBAC with induced labor onset and for another of the studies (64) the risk was only significantly raised for two out of the three definitions of hemorrhage the study considered. Two studies (44, 73) found a significantly reduced risk of hemorrhage for planned VBAC compared to ERCS (relative effect ranging from 0.22 to 0.34). The other seven studies (39, 44, 46, 53, 61, 62, 66, 72) found no significant difference in the risk (Figure 3).

##### Blood transfusion

**FIGURE 4:**
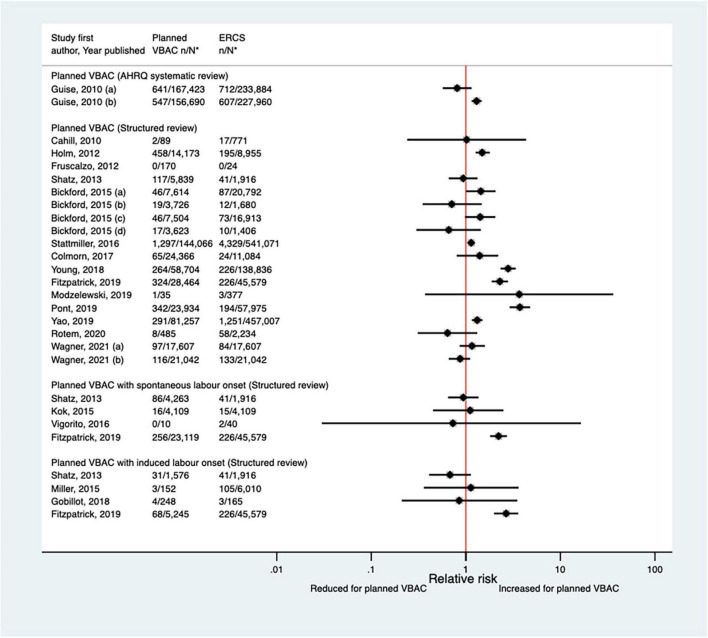
Risk of blood transfusion for planned VBAC vs. ERCS. *n, number in group with the outcome; *N*, Total number in group. Guise et al. (20) (a) risk in women who gave birth at any gestational age, (b) risk in women who gave birth at ≥ 37 weeks’ gestation; Bickford and Janssen (50) (a) risk in women with 1–2 prior CSs only, (b) risk in women with 1–2 prior CSs and ≥ 1 prior VB, (c) risk in women with 1 prior CS only, (d) risk in women with 1 prior CS and ≥ 1 prior VB. Wagner et al. (77) (a) Risk in women with no prior vaginal births. (b) Risk in women with ≥ 1 prior vaginal birth. AHRQ, Agency for Healthcare Research and quality; CS, cesarean section; ERCS, elective repeat cesarean section; VBAC, vaginal birth after previous cesarean; VB, vaginal birth.

Eighteen studies (35, 39, 41, 47, 50–52, 55, 57, 58, 62, 64, 65, 68–70, 72, 77) (10 population-based, 11 of women who gave birth at term) have investigated the occurrence of blood transfusion in relation to planned VBAC and ERCS since the AHRQ review (20). Reported absolute risks of blood transfusion in these studies varied from 0.00 to 3.23% for planned VBAC and 0.00–5.00% for ERCS. No cases of blood transfusion occurred in one small study (39), while seven of the larger studies (41, 50, 55, 64, 65, 69, 70) reported an elevated risk of blood transfusion for planned VBAC compared to ERCS (relative effect ranging from 1.14 to 3.73). However, for one of the studies (50), the elevated risk was only apparent amongst women without a prior vaginal birth. The remaining ten studies (35, 47, 51, 52, 57, 58, 62, 68, 72, 77) found no significant difference between planned VBAC and ERCS (Figure 4).

##### Maternal infection

**FIGURE 5:**
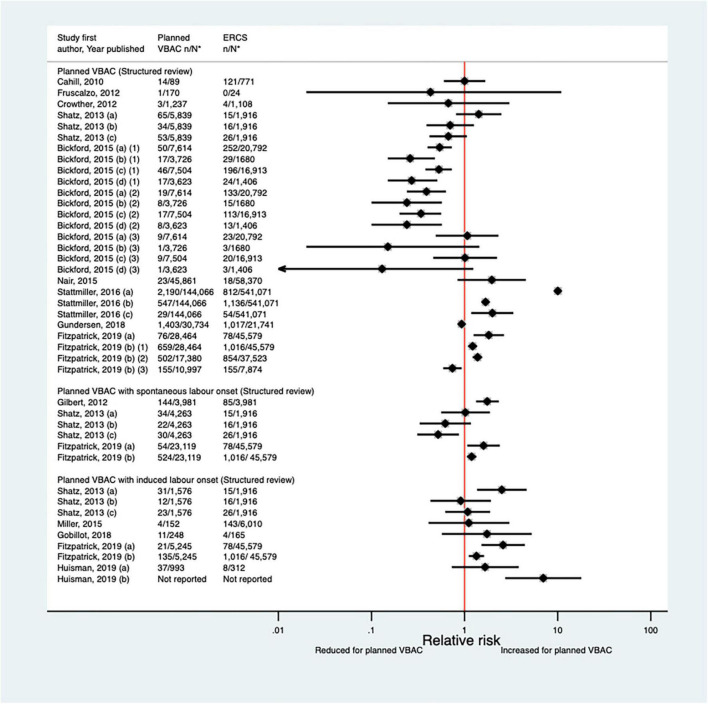
Risk of any type of maternal infection for planned VBAC vs. ERCS. *n, number in group with the outcome; *N*, Total number in group. Shatz et al. (47) (a) outcome: postpartum infection, (b) outcome: endometritis, (c) outcome: fever; Bickford and Janssen (50) (a) risk in women with 1–2 prior CSs only, (b) risk in women with 1–2 prior CSs and ≥ 1 prior VB, (c) risk in women with 1 prior CS only, (d) risk in women with 1 prior CS and ≥ 1 prior VB, (1) outcome: obstetric surgical wound infection, (2) outcome: puerperal infection, (3) outcome: puerperal sepsis; Stattmiller et al. (55) (a) outcome: chorioamnionitis, (b) outcome: major puerperal infection, (c) outcome: puerperal sepsis; Shatz et al. (47) (a) outcome: infection, (b) outcome: endometritis, (c) outcome: fever; Fitzpatrick et al. (65) (a) outcome: puerperal sepsis, (b) outcome: other puerperal infection, (1) risk in women with ≥ 1 prior CSs, (2) risk in women with ≥ 1 prior CSs and no prior VB, (3) risk in women with ≥ 1 prior CSs and ≥ 1 prior VB; Huisman et al. (66) (a) outcome: postpartum infection, defined as treated urinary tract infection, endometritis, pneumonia, wound infection or other unspecified suspected maternal infection (defined as fever of ≥ 38^°^C during labor or fetal tachycardia and start of broad-spectrum intravenous antibiotics for suspected infection), (b) outcome: fever during labor, defined as temperature ≥ 38^°^C. CS, cesarean section; ERCS, elective repeat cesarean section; VBAC, vaginal birth after previous cesarean; VB, vaginal birth.

Since the AHRQ review (20), a total of 13 studies (33, 35, 39, 40, 47, 50, 52, 53, 55, 62, 63, 65, 66) (five population-based, seven of women who gave birth at term) were identified as reporting on some form of maternal infectious morbidity. The type of infection reported amongst these studies varied widely and the criteria for infection was often not defined. Considering all definitions, absolute risks of infection ranged from 0.02 to 15.7% for planned VBAC and 0.00–15.7% for ERCS. When compared to ERCS, five studies (40, 47, 55, 65, 66) found that planned VBAC (one just with induced labor onset) was associated with an increased risk (relative effect 1.19–10.04) and three studies (47, 50, 65) found that planned VBAC (one just among women with a prior vaginal birth and one just with spontaneous labor onset) was associated with a reduced risk (relative effect 0.24–0.74) for at least one of the infection outcomes they examined. The remaining studies (33, 35, 39, 52, 53, 62, 63) reported no significant difference (Figure 5). Considering the specific type of infection, of the three studies (40, 47, 52) that reported on endometritis, one (40) found an increased risk of this outcome for planned VBAC compared to ERCS (aOR 1.75, 95% CI 1.33–2.33) while the others (47, 52) reported no significant difference. Only one study (55) reported on chorioamnionitis, finding the risk of this to be significantly higher risk for planned VBAC compared to ERCS (aOR 10.04, 95% CI 9.26–10.90). Of the five studies (35, 39, 47, 62, 66) that reported on fever, one (66) found an increased risk of this outcome for planned VBAC (with induced labor onset) compared to ERCS (aOR 7.00, 95% CI 2.73–17.95), one (47) found a reduced risk of this outcome for planned VBAC (with spontaneous labor onset, uRR 0.52, 95% CI 0.31–0.87), and the other studies (35, 39, 62) reported no significant difference. Wound infection was found to be significantly reduced for planned VBAC compared to ERCS in the one study (50) that examined this outcome (uRR as low as 0.26, 95% CI 0.15–0.48 in women with 1–2 prior cesarean sections and ≥ 1 prior vaginal birth). Considering maternal sepsis, while two studies (55, 65) found this to be significantly increased for planned VBAC compared to ERCS (relative effect ∼2), two studies (50, 53) found no significant difference.

##### Surgical injury

**FIGURE 6:**
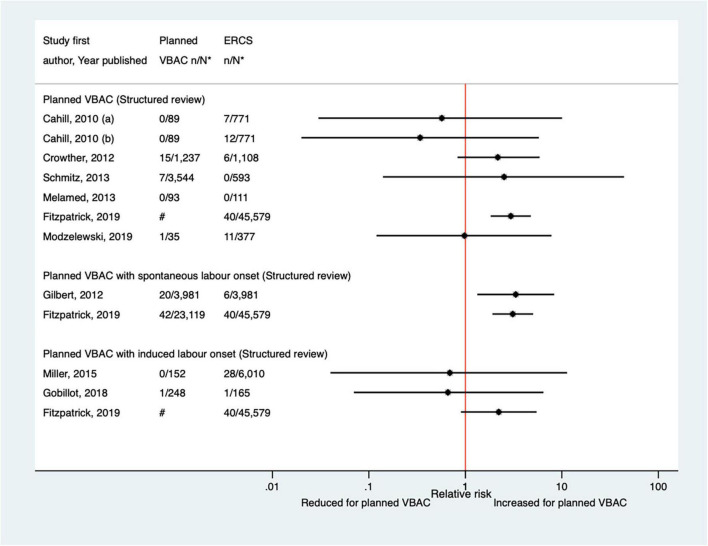
Risk of maternal surgical injury for planned VBAC vs. ERCS. *n, number in group with the outcome; *N*, Total number in group. Cahill et al. (35) (a) outcome: surgical injury, (b) outcome: bladder injury. ^#^Numbers have not been shown to protect against potential disclosure risks. ERCS, elective repeat cesarean section; VBAC, vaginal birth after previous cesarean.

The definition of surgical injury used by the nine predominately small studies (33, 35, 40, 44, 46, 52, 62, 65, 68) (one population-based, seven of women who gave birth at term) identified since the AHRQ review (20) varied and reported absolute risks of this outcome among the nine studies ranged from 0.00 to 2.86% for planned VBAC and 0.00–2.92% for ERCS. Only the two largest studies (40, 65) found a significant difference in the risk of surgical injury between planned VBAC and ERCS, reporting around a threefold increased risk for planned VBAC (with spontaneous labor onset) (Figure 6).

##### Length of hospital stay

Seven studies (33, 44, 62, 65, 66, 71, 72) have reported on the length of hospital stay since the AHRQ review (20). Five (33, 62, 65, 66, 71) of the studies found some evidence that the length of stay was shorter for planned VBAC compared to VBAC: two (33, 66) found the median length of postnatal stay was significantly shorter for planned VBAC (one with labor induction with a balloon catheter), although one of these found no significant difference in the percentage of women with a postnatal stay >7 days and the other reported no difference in the median total admission time; two (62, 71) found the mean or median length of hospitalization was shorter for planned VBAC (one with oxytocin induction); and the fifth study (65) found planned VBAC was associated with a reduced likelihood of having a postnatal hospital stay > 5 days, but only among women with one or more prior vaginal birth. Two other small studies (44, 72) found no significant difference in the proportion of women with a prolonged hospital stay (defined as either >5 or ≥ 7 days).

##### Breastfeeding

Only two studies (45, 65) (both population-based, one of women who gave birth at term) were identified as reporting on the effect of planned VBAC compared to ERCS on breastfeeding. One of the studies (45) reported that women giving birth by successful VBAC and those giving birth by cesarean section following an unsuccessful planned VBAC were both more likely to initiate breastfeeding than women giving birth by ERCS (66.6% vs. 58.9%, aOR 1.42, 95% CI 1.30–1.56 and 61.3% vs. 58.9%, aOR 1.15, 95% CI 1.01–1.31, respectively). The study did not report the risks for planned VBAC compared to ERCS but estimating this from the data provided suggests that women who had a planned VBAC were significantly more likely to initiate breastfeeding than women who gave birth by ERCS (65.1% vs. 58.9%, uRR 1.10, 95% CI 1.08–1.13). The second study (65) also found that women who planned a VBAC were significantly more likely to initiate breastfeeding and found they were more likely to breastfeed at ∼6-8 weeks postpartum [adjusted risk ratio (aRR) ∼1.2].

### Pelvic floor dysfunction/perineal trauma

Since the AHRQ review (20), only one study (33) has investigated the effect of planned VBAC compared to ERCS on perineal trauma. This study, which included a total of 2,345 women, reported that the risk of vulvar or perineal hematoma requiring evacuation was not significantly different for planned VBAC and ERCS (0.16% vs. 0.09%, aRR 1.79, 95% CI 0.16–20). Like the AHRQ review, no studies were identified that investigated the effect of planned VBAC compared to ERCS on the risk of urinary or fecal incontinence. However, a number of studies (79–83) (not included in this structured review) were identified that reported on the risk of obstetric anal sphincter injury in women who had a VBAC compared to primiparous women who had a vaginal birth, with most (79, 80, 82, 83) reporting an increased risk of this outcome in the women who had a VBAC.

#### Mental health

As maternal mental health was not included as an outcome in the AHRQ review (20), the current structured review searched the literature for relevant studies as far back as 1980. Only two studies (32, 75) investigating the effect of planned VBAC compared to ERCS on women’s mental health outcomes were identified. One of the studies (32), a small randomized controlled trial of 291 women recruited in a single hospital, used psychometric tests during pregnancy up until 6 months post-partum to measure woman’s anxiety, depression, and general psychological wellbeing. This study reported no significant differences in these measures between women who were randomized to planned VBAC compared to planned ERCS. The other study (75), a large population-based cohort study, reported that among women without a history of psychotropic drug use in the year before birth, planned VBAC compared to ERCS was associated with a 15% reduced risk of the mother being dispensed any psychotropic medication and a 17% reduced risk of the mother being dispensed antidepressants in the first year postpartum.

#### Baby/child outcomes

##### Perinatal and neonatal mortality

**FIGURE 7:**
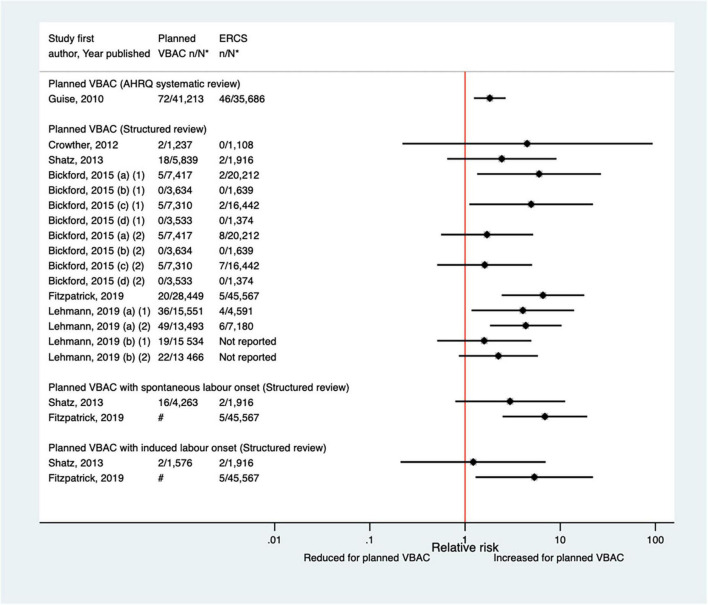
Risk of perinatal mortality for planned VBAC vs. ERCS. *n, number in group with the outcome; *N*, Total number in group; Bickford and Janssen (50) (a) risk in women with 1–2 prior CSs only, (b) risk in women with 1–2 prior CSs and ≥ 1 prior VB, (c) risk in women with 1 prior CS only, (d) risk in women with 1 prior CS and ≥ 1 prior VB, (1) outcome: intrapartum stillbirth or death at ≤ 7 days of birth, (2) outcome: intrapartum stillbirth or death up to 28 days of birth; Lehmann et al. (67) (a) outcome: intrapartum stillbirth, death within 28 days of birth, time of death unknown and pre-labor stillbirths delivered after 39 weeks’ gestation, (b) outcome: intrapartum stillbirth, death within 28 days of birth, and time of death unknown, (1) risk in “low-risk” women, (2) risk in “high-risk” women. ^#^Numbers have not been shown to protect against potential disclosure risks. AHRQ, Agency for Healthcare Research and quality; CS, cesarean section; ERCS, elective repeat cesarean section; VBAC, vaginal birth after previous cesarean; VB, vaginal birth.

Since the AHRQ review (20), five studies (33, 47, 50, 65, 67) (three population-based) were identified as reporting on perinatal mortality excluding congenital or lethal anomalies. One (47) of these studies did not restrict their study population to term born infants or attempt to control/take account of gestational age. The definition of perinatal mortality varied slightly among the five studies and the absolute risks of this outcome ranged from 0 to 3.8 per 1,000 for planned VBAC and 0–1.0 per 1,000 for ERCS. The largest three studies (50, 65, 67) found ∼5–7-fold significantly increased risk of perinatal mortality for planned VBAC compared to ERCS (one for intrapartum stillbirth and death within 7 days of birth, one for intrapartum stillbirth and death within 28 days of birth, and the other for intrapartum stillbirth, deaths within 28 days of birth, time of death unknown and prelabor stillbirths after 39 weeks’ gestation). The remaining rather small studies found no significant difference (33, 47) (Figure 7).

**FIGURE 8:**
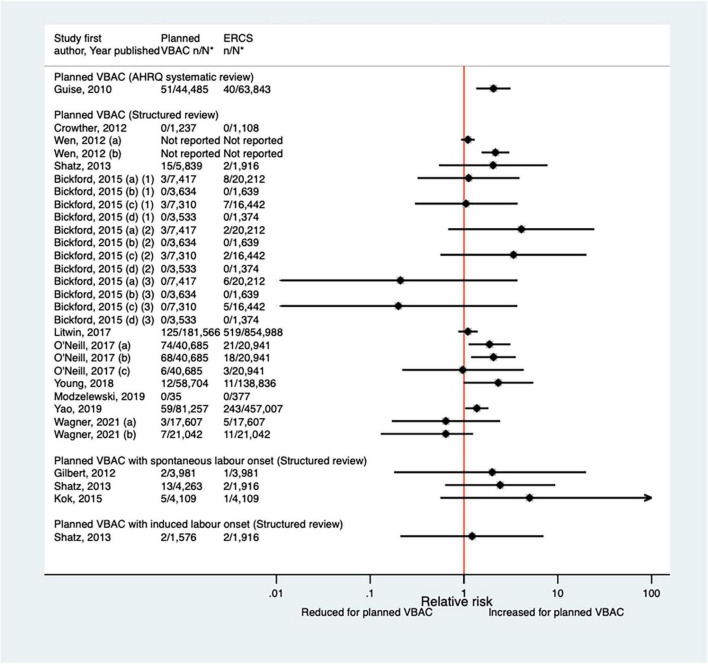
Risk of neonatal mortality for planned VBAC vs. ERCS. *n, number in group with the outcome; *N*, Total number in group. Wen et al. (42) (a) outcome: all cause neonatal death, (b) outcome: asphyxia associated neonatal death; Bickford and Janssen (50) (a) risk in women with 1–2 prior CSs only, (b) risk in women with 1–2 prior CSs and ≥ 1 prior VB, (c) risk in women with 1 prior CS only, (d) risk in women with 1 prior CS and ≥ 1 prior VB, (1) outcome: death at ≤ 28 days of birth, (2) outcome: death at ≤ 7 days of birth, (3) outcome: death at 8–28 days of birth; O’Neill et al. (60) (a) outcome: death at ≤ 28 days of birth, (b) outcome: death at ≤ 7 days of birth, (c) outcome: death at 8–28 days of birth. Wagner et al. (77) (a) Risk in women with no prior vaginal births. (b) Risk in women with ≥ 1 prior vaginal birth. AHRQ, Agency for Healthcare Research and quality; CS, cesarean section; ERCS, elective repeat cesarean section; VBAC, vaginal birth after previous cesarean; VB, vaginal birth.

Twelve studies (33, 40, 42, 47, 50, 51, 59, 60, 64, 68, 70, 77) (eight population-based) were identified as reporting on neonatal mortality excluding congenital or lethal anomalies since the AHRQ review (20). Only one (47) of these studies did not restrict their study population to term born infants or attempt to control/take account of gestational age. Neonatal mortality was not defined in six of the studies (40, 42, 47, 64, 68, 70) while definitions varied among the other studies. Absolute risks of neonatal mortality ranged from 0 to 3.0 per 1,000 for planned VBAC and 0–1.0 per 1,000 for ERCS with only three of the studies (42, 60, 70) reporting a significant difference in the risk between planned VBAC and ERCS; these studies found ∼1.4–2-fold significantly increased risk of neonatal mortality for planned VBAC compared to ERCS, although for one of the studies (42) the elevated risk was only apparent for asphyxia associated neonatal death rather than all cause neonatal death and for another of the studies (60) the increased risk was confined to early (≤7 days of birth) rather than late (8–28 days of birth) neonatal death (Figure 8).

##### Neonatal respiratory intervention/morbidity

**FIGURE 9:**
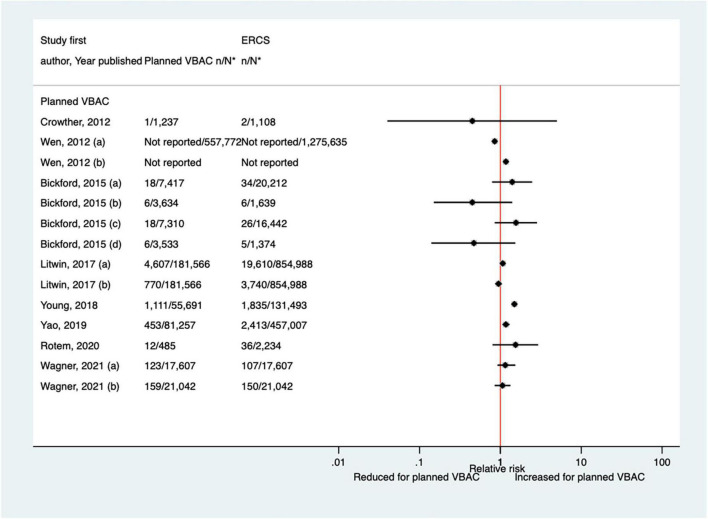
Risk of neonatal ventilation for planned VBAC vs. ERCS. *n, number in group with the outcome; *N*, Total number in group. Wen et al. (42) (a) risk in total study population, (b) risk in infants born to “low-risk” women without pre-existing medical problems, pregnancy complications, preterm birth or an infant with low birth weight or congenital anomaly. Bickford and Janssen (50) (a) risk in women with 1–2 prior CSs only, (b) risk in women with 1–2 prior CSs and ≥ 1 prior VB, (c) risk in women with 1 prior CS only, (d) risk in women with 1 prior CS and ≥ 1 prior VB. Litwin et al. (59) (a) outcome: assisted ventilation, (b) outcome: assisted ventilation > 6 h. Wagner et al. (77) (a) Risk in women with no prior vaginal births. (b) Risk in women with ≥ 1 prior vaginal birth. CS, cesarean section; ERCS, elective repeat cesarean section; VBAC, vaginal birth after previous cesarean; VB, vaginal birth.

Since the AHRQ review (20) a total of 12 studies (33, 40, 42, 46, 50, 51, 59, 64, 65, 70, 72, 77) (eight population-based) have reported on some form of neonatal respiratory intervention/morbidity. Two of the studies (42, 72) did not confine their study population to term infants, although one (42) did conduct a sub-group analysis on “low-risk” women which excluded those who gave birth preterm. Eight of the studies (33, 42, 50, 59, 64, 70, 72, 77) investigated the need for some form of ventilation, reporting absolute risks ranging from 0.08 to 2.54% for planned VBAC and 0.16–2.29% for ERCS. Four of these studies (42, 59, 64, 70) found ∼1.1–1.2-fold increased risk of this outcome for planned VBAC compared to ERCS, although for one of the studies (42) the increased risk was only apparent among women considered to be “low-risk” and for another of the studies (57) no significant difference was found when ventilation > 6 h was considered. The other four studies (33, 50, 72, 77) reported no significant difference in the risk of ventilation between planned VBAC and ERCS (Figure 9). The need for intubation was investigated in three of the studies (46, 64, 65) with absolute risks of this outcome ranging from 0.65 to 2.00% for planned VBAC and 0.3–1.4% for ERCS. The two largest studies (64, 65) reported a significantly increased risk (relative effect ranging from ∼1.5 to 6) for planned VBAC compared to ERCS (one for ventilation with endotracheal intubation excluding ventilation requiring continuous positive air-way pressure and one for resuscitation requiring drugs and/or intubation) and the remaining rather small study finding no significant difference (46). Three studies (40, 51, 72) investigated transient tachypnea of the newborn, with absolute risks of this outcome ranging from 0.66 to 2.69% for planned VBAC group and 1.10–2.99% for ERCS. One (51) of the studies reported a reduced risk of this outcome for the planned VBAC group (aOR 0.59, 95% CI 0.36–1.00) and the other studies (40, 72) found no significant difference.

##### Hypoxic-ischemic encephalopathy/asphyxia

**FIGURE 10:**
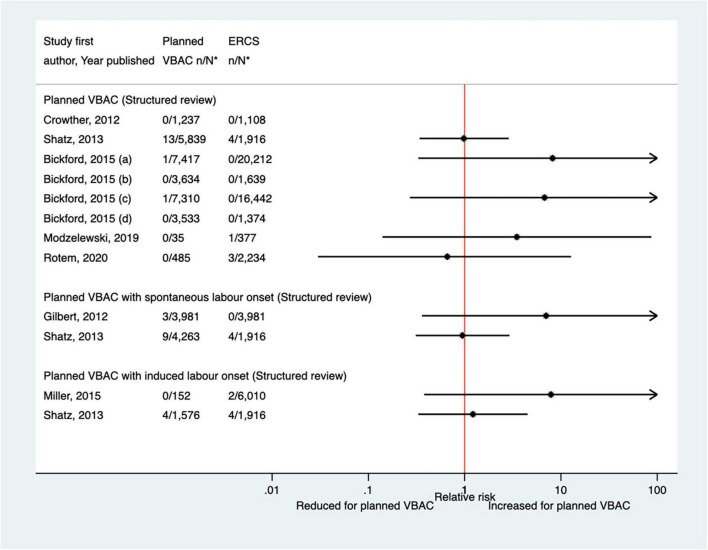
Risk of hypoxic–ischemic encephalopathy/asphyxia for planned VBAC vs. ERCS. *n, number in group with the outcome; *N*, Total number in group. Bickford and Janssen (50) (a) risk in women with 1–2 prior CSs only, (b) risk in women with 1–2 prior CSs and ≥ 1 prior VB, (c) risk in women with 1 prior CS only, (d) risk in women with 1 prior CS and ≥ 1 prior VB. CS, cesarean section; ERCS, elective repeat cesarean section; VBAC, vaginal birth after previous cesarean; VB, vaginal birth.

**FIGURE 11:**
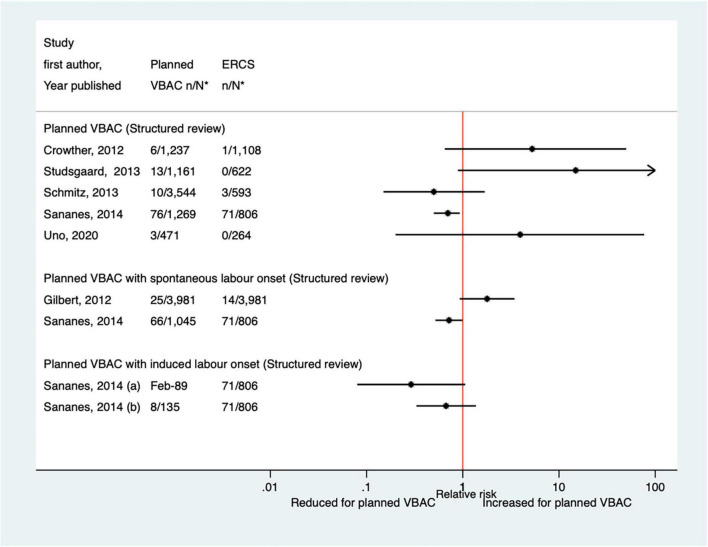
Risk of cord pH < 7 for planned VBAC vs. ERCS. *n, number in group with the outcome; *N*, Total number in group. Sananes et al. (49) (a) planned VBAC with induction of labor with oxytocin and amniotomy, (b) planned VBAC with induction of labor with foley catheter. ERCS, elective repeat cesarean section; VBAC, vaginal birth after previous cesarean.

Thirteen predominately small studies (33, 40, 46–50, 52, 62, 66, 68, 72, 74) (one population-based) were identified as attempting to investigate hypoxic-ischemic encephalopathy or asphyxia in some way since the AHRQ review (20). Five of these studies (47, 49, 52, 72, 74) did not restrict their study population to term infants and made no attempt to control for gestational age. Seven of the studies (33, 40, 47, 50, 52, 68, 72) reported on hypoxic-ischemic encephalopathy/asphyxia without defining it, with all finding no significant difference in the risk of this outcome between planned VBAC and ERCS (Figure 10). Of the six studies (33, 40, 46, 48, 49, 74) that investigated the proportion of infants with a cord pH of less than 7 (an indication of hypoxic–ischemic encephalopathy/asphyxia), one (49) found this to be significantly reduced (uRR ∼0.7) for planned VBAC compared to ERCS (except where planned VBAC had induced labor onset) while the others reported no significant difference (Figure 11). One small study (66) found no significant difference between the planned VBAC and ERCS groups in the proportion of infants with a cord pH of less than 7.10, while another small study (62) found ∼5-fold increased risk of infants having a cord pH <7.15 in the planned VBAC group (with oxytocin labor induction).

##### Neonatal sepsis/infection

Four studies (33, 40, 51, 68) (one population-based) were identified as reporting on neonatal sepsis/infection since the AHRQ review (20). All restricted their study population to infants born at term. Absolute risks of neonatal sepsis/infection ranged from 0.00 to 5.30% for planned VBAC and 0.00–3.11% for ERCS. One of the studies (40) grouped suspected and confirmed sepsis (without defining it), finding an increased risk of this outcome for planned VBAC compared to ERCS (aOR 1.72, 95% CI 1.39–2.17). The other three studies considered “proven” systemic infection (33), neonatal infection (51) and neonatal sepsis (68) (none defined), respectively, finding no significant difference between planned VBAC and ERCS or reporting no cases.

##### Birth trauma

**FIGURE 12:**
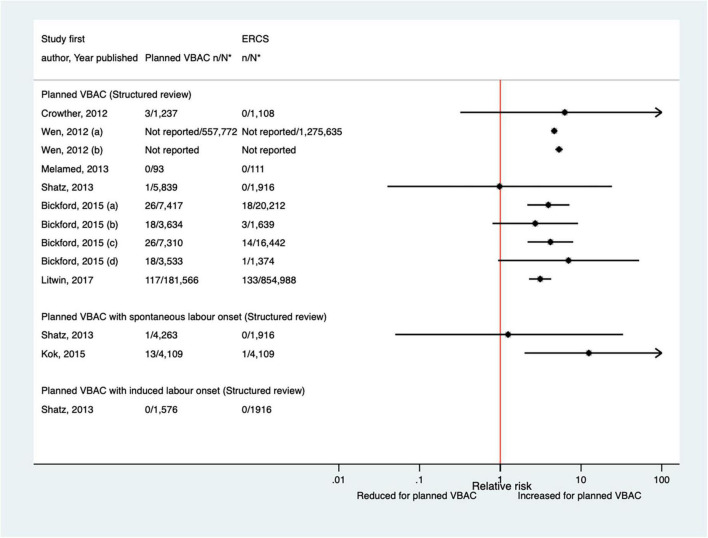
Risk of birth trauma for planned VBAC vs. ERCS. *n, number in group with the outcome; *N*, Total number in group. Wen et al. (42) (a) risk in total study population, (b) risk in infants born to “low-risk” women without pre-existing medical problems, pregnancy complications, preterm birth or an infant with low birth weight or congenital anomaly; Bickford and Janssen (50) (a) risk in women with 1–2 prior CSs only, (b) risk in women with 1–2 prior CSs and ≥ 1 prior VB, (c) risk in women with 1 prior CS only, (d) risk in women with 1 prior CS and ≥ 1 prior VB. CS, cesarean section; ERCS, elective repeat cesarean section; VBAC, vaginal birth after previous cesarean; VB, vaginal birth.

Of the seven studies (33, 42, 44, 47, 50, 51, 59) (four population-based) that have investigated birth trauma since the AHRQ review (20), only one (47) did not restrict their study population to term born infants or attempt to control/take account of gestational age. The definition of birth trauma used by the seven studies varied and absolute risks ranged from 0.00 to 0.51% for planned VBAC and 0.00–0.18% for ERCS. While the four largest studies (42, 50, 51, 59) reported an increased risk of birth trauma for planned VBAC compared to ERCS (RRs ranging from 3.12 to 12.50), the remaining rather small studies reported no significant difference (33, 47) or no cases at all of birth trauma (44) (Figure 12). However, one of the studies found that the risk was only significantly elevated amongst women without a prior vaginal birth (50).

##### Admission to neonatal intensive care unit

**FIGURE 13:**
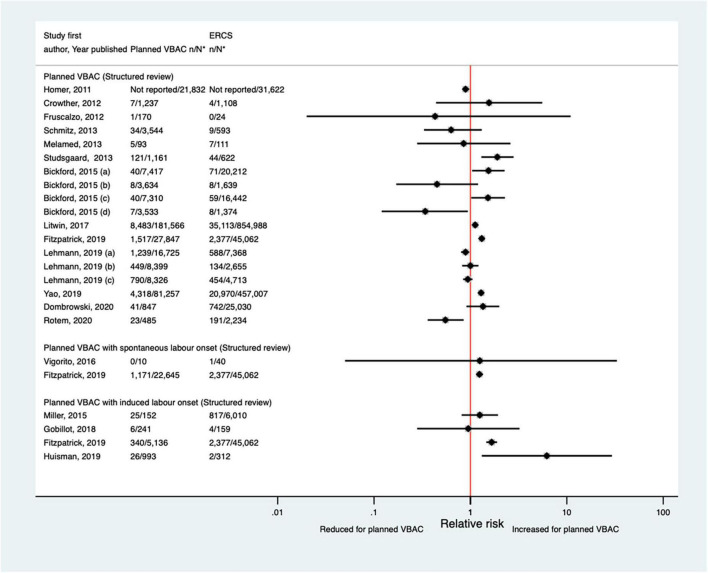
Risk of NICU admission for planned VBAC vs. ERCS. *n, number in group with the outcome; *N*, Total number in group. Bickford and Janssen (50) (a) risk in women with 1–2 prior CSs only, (b) risk in women with 1–2 prior CSs and ≥ 1 prior VB, (c) risk in women with 1 prior CS only, (d) risk in women with 1 prior CS and ≥ 1 prior VB; Lehmann et al. (67) (a) risk in all of study population, (b) risk in “low-risk” women, (c) risk in “high-risk” women. CS, cesarean section; ERCS, elective repeat cesarean section; NICU, neonatal intensive care unit; VBAC, vaginal birth after previous cesarean; VB, vaginal birth.

Of the 17 studies (33, 38, 39, 44, 46, 48, 50, 52, 57, 59, 62, 65–67, 70–72) (seven population-based) that have examined NICU admission since the AHRQ review (20), four (39, 52, 57, 72) did not confine their study population to term born infants and made no attempt to control for gestational age. None of the studies described the NICU admission criteria, the reason for admission was only reported in one study (50), and only one study (33) gave any information regarding the length of stay. Absolute risks of NICU admission varied from 0.00 to 18.7% for planned VBAC and 0.00–13.6% for ERCS and studies reported mixed results when risks were compared for planned VBAC and ERCS. While three studies found a reduced risk (38, 67, 72) (relative effect ranging from 0.55 to 0.89), six studies (48, 50, 59, 65, 66, 70) reported an elevated risk of NICU admission for planned VBAC compared to ERCS (RRs ranging from 1.12 to 6.20), although in one of these studies (50) the increased risk was confined to infants born to women who had not had a prior vaginal birth. The other eight predominately smaller studies reported no significant difference between planned VBAC and ERCS (33, 39, 44, 46, 52, 57, 62, 71) (Figure 13).

##### Apgar score

**FIGURE 14:**
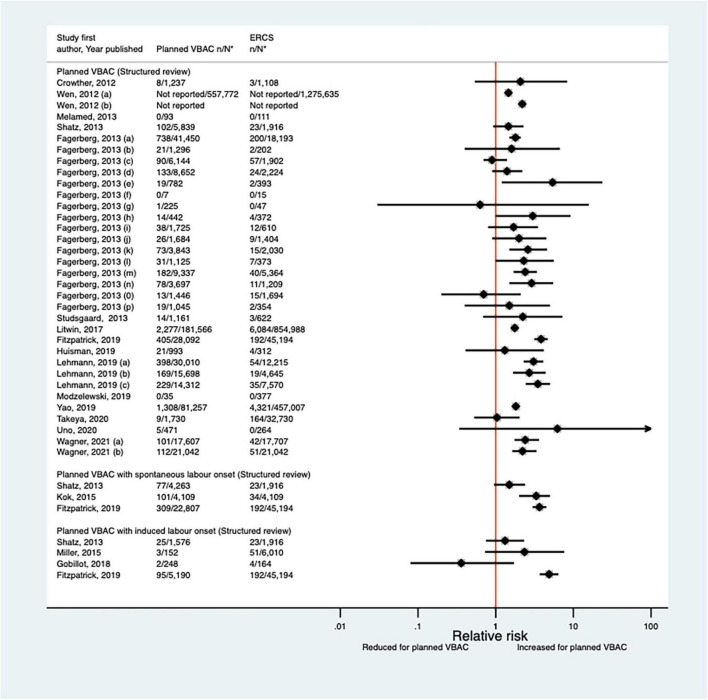
Risk of Apgar score < 7 at 5 min after birth for planned VBAC vs. ERCS. *n, number in group with the outcome; *N*, Total number in group. Wen et al. (42) (a) risk in total study population, (b) risk in infants born to “low-risk” women without pre-existing medical problems, pregnancy complications, preterm birth or an infant with low birth weight or congenital anomaly; Fagerberg et al. (43) (a) risk in all women with one prior CS, (b) risk in women whose indication for first CS multiple gestation, (c) risk in women whose indication for first CS preterm birth, (d) risk in women whose indication for first CS breech or other malpresentation ≥ 37 weeks’ gestation, (e) risk in women whose indication for first CS significant congenital malformation, (f) risk in women whose indication for first CS rupture of uterus, (g) risk in women whose indication for first CS placenta praevia, (h) risk in women whose indication for first CS diabetes mellitus/gestational diabetes, (i) risk in women whose indication for first CS small for gestational age/suspected intrauterine growth restriction, (j) risk in women whose indication for first CS large for gestational age/suspected large for gestational age/birth weight > 4,500 g, (k) risk in women whose indication for first CS prolonged pregnancy ≥ 42 weeks’ gestation, (l) risk in women whose indication for first CS severe pregnancy complications/severe maternal disease, (m) risk in women whose indication for first CS complications during labor/birth, (n) risk in women whose indication for first CS fetal distress/death unexplained by indications above, (o) risk in women whose indication for first CS no indication listed above/mild conditions not classified elsewhere, (p) risk in women who had no diagnosis available for indication for first CS; Lehmann et al. (67) (a) risk in all of study population, (b) risk in “low-risk” women, (c) risk in “high-risk” women. Wagner et al. (77) (a) Risk in women with no prior vaginal births. (b) Risk in women with ≥ 1 prior vaginal birth. CS, cesarean section; ERCS, elective repeat cesarean section; VBAC, vaginal birth after previous cesarean.

**FIGURE 15:**
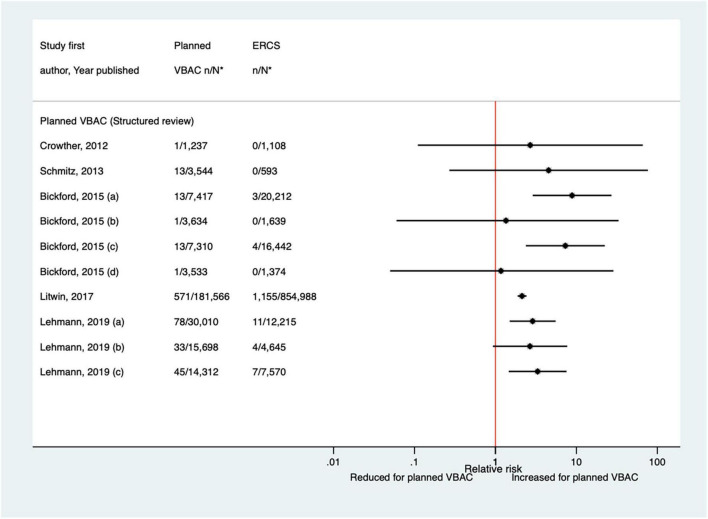
Risk of Apgar score < 4 at 5 min after birth for planned VBAC vs. ERCS. *n, number in group with the outcome; *N*, Total number in group. Bickford and Janssen (50) (a) risk in women with 1–2 prior CSs only, (b) risk in women with 1–2 prior CSs and ≥ 1 prior VB, (c) risk in women with 1 prior CS only, (d) risk in women with 1 prior CS and ≥ 1 prior VB; Lehmann et al. (67) (a) risk in all of study population, (b) risk in “low-risk” women, (c) risk in “high-risk” women. CS, cesarean section; ERCS, elective repeat cesarean section; VBAC, vaginal birth after previous cesarean; VB, vaginal birth.

Since the AHRQ review (20) 20 studies (33, 42–44, 46–48, 50–52, 59, 62, 65–68, 70, 73, 74, 77) (nine population-based) were identified since the AHRQ review as reporting on Apgar score in relation to infants born by planned VBAC vs. ERCS. Three of these studies (46, 52, 74) did not restrict their study population to term born infants or attempt to take account of gestational age. Of the 18 studies that reported on the proportion of infants with a 5-min Apgar score of <7, absolute risks of this outcome ranged from 0.00 to 3.17% for planned VBAC and 0.00–3.00% for ERCS, with eight of the studies (42, 43, 51, 59, 65, 67, 70, 77) finding an elevated risk associated with planned VBAC compared to ERCS (relative effect ranging from 1.46 to 5.40). However, for one of the studies (43) the increased risk was only apparent for the overall study population and for infants born to women who had certain indications for their first cesarean, with the risk reported to vary greatly according to indication for the first cesarean. The remaining predominately smaller studies reported no significant difference (33, 47, 48, 52, 62, 66, 73, 74) or reported that there were no infants in their study with an Apgar score of <7 at 5 min (44, 68) (Figure 14). Of the five studies (33, 46, 50, 59, 67) that reported on the proportion of infants with a 5-min Apgar score of <4, absolute risks of this outcome ranged from 0.03 to 0.37% for planned VBAC and 0.00–0.14% for ERCS with only the three largest studies (50, 59, 67) findings a significantly higher risk (relative effect ranging from 2.13 to 8.85) for planned VBAC compared to ERCS and the other studies (33, 46) reporting no difference (Figure 15). However, one of the studies (50) reported that the risk was only significantly elevated among women without a prior vaginal birth and another of the studies (67) reported that the risk was only significantly increased among infants born to “high-risk” women.

##### Neurodevelopment

Two population-based studies (54, 76), both conducted in Scotland, were the only studies identified that assessed the effect of planned VBAC compared to ERCS on the child’s neurological development. One of the studies (54) reported that there was no significant difference in the risk of learning disability or cerebral palsy among around 8,000 singleton school-aged children born at term following a planned VBAC compared to an ERCS, although this study did find an increased risk of learning disability associated with repeat cesarean after an unsuccessful VBAC compared to successful VBAC (3.7% vs. 2.3%, aOR 1.64, 95% CI 1.17–2.29). The other study (76) of nearly 45,000 children found little evidence of an association between planned mode of birth after previous cesarean section and special educational needs in childhood (age 4–11 years) beyond a small absolute increased risk of sensory impairment seen for planned VBAC with labor induction compared to ERCS (1.18% vs. 0.78%, aOR 1.60, 95% CI 1.09–2.34) that may be the result of performing multiple comparisons or residual confounding.

##### Health problems in childhood

The AHRQ review (20) did not include childhood health problems and so the current structured review searched the literature for relevant studies that included this outcome as far back as 1980. This identified just one study, a population-based study of second-born singleton children born at term conducted in Scotland (54). This study found no significant difference in the risk of various adverse childhood health outcomes between planned VBAC and ERCS, including obesity at age 5 years, salbutamol inhaler prescription (proxy for asthma) at age 5 years or type 1 diabetes mellitus, cancer, hospitalization with asthma or hospitalization with inflammatory bowel disease, where follow-up time was up to 21 years. On the other hand, the risk of hospitalization with asthma, but not the other health outcomes, was found to be significantly raised for children born by repeat cesarean section after an unsuccessful VBAC and for children born by ERCS when compared to those born by VBAC [adjusted hazard ratio (aHR) 1.18, 95% CI 1.05–1.33 and aHR 1.24, 95% CI 1.09–1.42 respectively]. The power of the study was considered adequate to examine common outcomes such as obesity, although a fifth of children were missing data on obesity, which as the authors acknowledged, may have biased these findings.

## Discussion

This review found some evidence that planned VBAC compared to ERCS is associated with a lower risk of maternal mortality, a shorter length of hospital stay, and a higher likelihood of breastfeeding, but also an increased risk of serious maternal complications such as uterine rupture, as well as a higher risk of perinatal/neonatal mortality and some types of neonatal morbidity. However, the absolute risk of adverse outcomes in the perinatal period appears to be small for either birth approach. Furthermore, the limited evidence available suggests that planned mode of birth after previous cesarean section is not associated with the child’s subsequent risk of experiencing adverse neurodevelopmental or health problems in childhood. While this review provides valuable insight into the associated outcomes of planned VBAC and ERCS for women and their children, several limitations and gaps with the evidence were apparent.

Key limitations highlighted in the AHRQ systematic review (20) were still evident with much of the subsequent literature, including questionable comparability between the groups (including women in the ERCS group who were not eligible to attempt planned VBAC) and inferring intended mode of birth from actual mode of birth resulting in misclassification of women who intended ERCS but went into spontaneous labor before their cesarean or women who intended planned VBAC but gave birth by cesarean. Due to few studies, inadequate or variable outcome definition, inconsistency in results between studies or a lack of precision in estimates of effect, a particular lack of robust evidence was still apparent for a whole range of outcomes. This included the following outcomes: maternal hemorrhage, maternal infection, breastfeeding initiation or continuation, maternal pelvic floor dysfunction/perineal trauma, women’s mental health, neonatal respiratory intervention/morbidity, hypoxic–ischemic encephalopathy/asphyxia, neonatal sepsis, admission to a NICU, child’s neurological development, and health problems in childhood. Evidence on the longer-term outcomes for women and their children was particularly sparse.

Much of the literature identified by the AHRQ systematic review (20) consisted of non-population-based cohort studies conducted in a single or small number of mainly tertiary or academic medical institutions. Such studies tend to be prone to several limitations such as limited generalizability and lack of statistical power. Although more population-based studies have been conducted since the AHRQ review much of the literature still consists of rather small studies that are likely to lack statistical power, particularly given the rarity of many of the outcomes considered. Although a large randomized controlled trial would arguably be the gold standard methodology for assessing the effects of planned VBAC compared to ERCS, most of the literature also still consists of non-randomized studies with only two small trials identified. One of these trials included around 300 women and only investigated the effects on women’s psychological health (32), while only 22 women consented to randomization of planned mode of birth in the other trial (33). The latter study suggests that a large randomized controlled trial in this area is unlikely to be feasible as few women are likely to consent to participate. Consequently, high quality observational studies offer the best opportunity to further inform the evidence in this area. Unfortunately, in the existing observational studies risk estimates were often not adjusted, or only minimally adjusted, for confounding factors such that any observed effects may be due to differences between the planned VBAC and ERCS groups. Furthermore, studies seldom investigated what factors may modify any effects of mode of birth. Knowing whether there are certain subgroups (such as women without a prior vaginal birth) who have an increased or reduced risk of experiencing an adverse effect of mode of birth would be useful for the counseling of pregnant women with a prior cesarean.

Further limitations with the existing literature included the fact that most of the studies did not distinguish between whether planned VBAC was attempted with or without labor induction and/or augmentation. This distinction is important as labor induction/augmentation in women with previous cesarean has been associated with an increased risk of certain complications such as uterine rupture as well as a reduced likelihood of achieving a successful VBAC (84–88). It has also been suggested that synthetic oxytocin used for labor induction and/or augmentation may reach the neonate’s brain and desensitize oxytocin receptors, possibly leading to adverse neurodevelopmental effects (89). Another limitation with the literature includes the fact that the recruitment period of around a third of the studies extended back to the 1980s or 1990s. Findings from such studies may not be relevant to current populations owing to advances in obstetric and neonatal care and changes in clinical practice and maternal characteristics (1, 90, 91) since this time. Some studies were also limited by the fact that they did not restrict their study population to singleton and/or term births and made no attempt to control for plurality and/or gestational age. Women with a singleton pregnancy at term who have a history of previous cesarean section are the main group of women UK guidelines (7, 8) recommend are candidates for planned VBAC or ERCS and should be counseled about both options. Furthermore, the risk of adverse outcomes is likely to differ between singleton and multiple births (92, 93), and between term and non-term births (94, 95).

This structured review has a number of strengths and limitations. Strengths include the fact that a structured explicit approach was used to search three databases for relevant studies meeting pre-specified eligibility criteria, supplemented by searching of reference lists. Our review also considered a wide range of both maternal and baby/child outcomes. However, article screening and data extraction were performed by a single author and the results were limited to English-language papers, which may have increased the chance of potentially relevant papers being overlooked. Also, in contrast to what is typically done in a full systematic review, no formal evaluation of the risk of reporting bias or assessment/rating of the quality of the included studies was performed. It is important to highlight that our review did not consider all possible outcomes. Most significantly, it did not include complications in subsequent pregnancies. Cesareans have been linked to an increased risk of various complications in future pregnancies. The latest Royal College of Obstetricians and Gynecologists (RCOG) guidelines (7) state that there is considerable data in particular to show that repeated ERCS is associated with an increased risk of placenta praevia, morbidly adherent placenta, and surgical complications such as hysterectomy in subsequent pregnancies/births.

## Conclusion and implications

This review suggests that while planned VBAC compared to ERCS is associated with an increased risk of various serious birth-related complications for both the mother and her baby, the absolute risk of these complications is small for either birth approach. This review also found some evidence that planned VBAC compared to ERCS is associated with benefits such as a shorter length of hospital stay and a higher likelihood of breastfeeding. The limited evidence available also suggests that planned mode of birth after previous cesarean section is not associated with the child’s subsequent risk of experiencing adverse neurodevelopmental or health problems in childhood. This information can be used to manage and counsel women with previous cesarean section about their subsequent birth choices. Collectively, the evidence supports existing consensus that there are risks and benefits associated with both planned VBAC and ERCS, and therefore women without contraindications to VBAC should be given an informed choice about planned mode of birth after previous cesarean section (7–11). However, further studies into the longer-term effects of planned mode of birth after previous cesarean section are needed along with more research to address the other key limitations and gaps that have been highlighted with the existing evidence.

## Author contributions

KF conceived the study, gained funding, performed the review, and wrote the first draft of the manuscript. MQ and JK supervised the study. All authors contributed to the design and interpretation of the study, critically reviewed the article, and approved the final version for publication.
