# Supplementary material for: Planned mode of birth after previous cesarean section: A structured review of the evidence on the associated outcomes for women and their children in high-income setting
Source: Front Med (Lausanne). 2022 Sep 6;9:920647. doi: 10.3389/fmed.2022.920647 (PMC9486480; doi:10.3389/fmed.2022.920647)
Supplement: Supplementary file 1 [file Data_Sheet_1.docx]

## Supplementary material

## Literature review - databases and search terms

| 1. exp Vaginal Birth after Cesarean/  2. vbac.mp.  3. (vagina$ adj3 (birth$ or born or  deliver$) adj7 ((after$ or follow$ or  previous$ or prior or history) adj3  (cesarean$ or caesarean$))).mp.  4. 1 or 2 or 3  5. exp "Trial of Labor"/  6. (trial of labor or trial of labour).mp.  7. tol.mp.  8. 6 or 7  9. exp pregnancy/ or exp pregnancy  complications/  10. exp Infant, Newborn/  11. exp child/  12. exp Obstetrics/  13. exp Obstetric Surgical Procedures/  14. 9 or 10 or 11 or 12 or 13  15. 8 and 14  16. 15 or 5  17. exp Cesarean Section, Repeat/  18. ((repeat$ or multip$ or another or  prior or previous$) adj3 (cesarean$  or caesarean$)).mp.  19. ((second or third) adj cesarean).mp.  20. 17 or 18 or 19  21. 4 or 16 or 20  22. limit 21 to (english language and  yr=”2009-Current”)  23. exp animals/ not humans.sh.  24. 22 not 23 | 1. exp Vaginal Birth after Cesarean/  2. vbac.mp.  3. (vagina$ adj3 (birth$ or born or  deliver$) adj7 ((after$ or follow$ or  previous$ or prior or history) adj3  (cesarean$ or caesarean$))).mp.  4. 1 or 2 or 3  5. exp "Trial of Labor"/  6. (trial of labor or trial of labour).mp.  7. tol.mp.  8. 5 or 6 or 7  9. exp Cesarean Section, Repeat/  10. ((repeat$ or multip$ or another or prior  or previous$) adj3 (cesarean$ or  caesarean$)).mp.  11. ((second or third) adj cesarean).mp.  12. 9 or 10 or 11  13. 4 or 8 or 12  14. Mental disorders/ or Mood Disorders/  or Depressive disorder/ or Depression,  Postpartum/ or Depressive disorder,  major/ or Depressive disorder,  treatment-resistant/ or Dysthymic  Disorder/ or exp Anxiety Disorders/ or  anxiety/ or Stress Disorders, Post-  Traumatic/ or exp "bipolar and related  disorders"/ or exp "schizophrenia  spectrum and other psychotic  disorders"/ or exp Psychotropic Drugs/  15. (mental health or mental illness or  psychological health or depression or  anxiety or post traumatic stress or  Post-traumatic stress or bipolar or  Schizophrenia or psychoses or  Psychosis or psychotic or  antidepressant or antipsychotic or  anxiolytics or antimanic or  psychotropic).mp.  16. 14 or 15  17. 13 and 16  18. limit 17 to (English language and  yr=”1980 – 2008”)  19. exp animals/ not humans.sh.  20. 18 not 19 | 1. exp Vaginal Birth after Cesarean/  2. vbac.mp. [mp=title, original title, abstract,  name of substance word, subject heading word]  3. (vagina$ adj3 (birth$ or born or deliver$) adj7  ((after$ or follow$ or previous$ or prior or  history) adj3 (cesarean$ or caesarean$))).mp.  4. 1 or 3 or 2  5. exp "Trial of Labor"/  6. (trial of labor or trial of labour).mp.  7. tol.mp.  8. 6 or 7  9. exp pregnancy/ or exp pregnancy complications/  10. exp Infant, Newborn/  11. exp child/  12. exp Obstetrics/  13. exp Obstetric Surgical Procedures/  14. 9 or 10 or 11 or 12 or 13  15. 8 and 14  16. 15 or 5  17. exp Cesarean Section, Repeat/  18. ((repeat$ or multip$ or another or prior or  previous$) adj3 (cesarean$ or caesarean$)).mp.  19. ((second or third) adj cesarean).mp.  20. 17 or 18 or 19  21. 4 or 16 or 20  22. Pediatric Obesity/  23. ((child* or pediatric or paediatric) adj3  obes*).mp.  24. exp Neoplasms/  25. (leukaemia or leukemia or lymphoma or  sarcoma or Osteosarcoma or Neuroblastoma  or Retinoblastoma or Rhabdomyosarcoma or  cancer* or tumor* or tumour*).mp.  26. exp Infection/ or exp respiratory tract infection/  or exp Pneumonia/ or exp Influenza, Human/  or exp Bronchitis/ or exp Diarrhea/ or exp  Gastroenteritis/ or exp Meningitis/ or Bacterial  Infections/  27. (infection or infectious or respiratory or  pneumonia or influenza or bronchitis or  bronchiolitis or gastroenteritis or diarrhea or  diarrhoea or meningitis or viral or bacterial).mp.  28. exp Diabetes Mellitus, Type 1/  29. (type 1 diabetes or type i diabetes).mp.  30. Asthma/  31. asthma*.mp.  32. exp Inflammatory Bowel Diseases/  33. (inflammatory bowel disease or crohn* disease  or colitis).mp.  34. 24 or 25 or 26 or 27 or 28 or 29 or 30 or 31 or  32 or 33  35. 21 and 34  36. limit 35 to "all child (0 to 18 years)"  37. 21 and (22 or 23)  38. 36 or 37  39. limit 38 to (english language and yr="1980 –  2008") |
| --- | --- | --- |

**- MEDLINE:**

| 1. exp Vaginal Birth after Cesarean/  2. vbac.mp.  3. (vagina$ adj3 (birth$ or born or  deliver$) adj7 ((after$ or follow$ or  previous$ or prior or history) adj3  (cesarean$ or caesarean$))).mp.  4. 1 or 2 or 3  5. exp "Trial of Labor"/  6. (trial of labor or trial of labour).mp.  7. tol.mp.  8. 6 or 7  9. exp pregnancy/ or exp pregnancy  complications/  10. exp Infant, Newborn/  11. exp child/  12. exp Obstetrics/  13. exp Obstetric Operation/  14. 9 or 10 or 11 or 12 or 13  15. 8 and 14  16. 15 or 5  17. exp Cesarean Section, Repeat/  18. ((repeat$ or multip$ or another or  prior or previous$) adj3 (cesarean$  or caesarean$)).mp.  19. ((second or third) adj cesarean).mp.  20. 17 or 18 or 19  21. 4 or 16 or 20  22. limit 21 to (english language and  yr=”2009-Current”)  23. (exp animals/ or nonhuman/) not  human/  24. 22 not 23  25. conference*.pt  26. 24 not 25 | 1. exp Vaginal Birth after Cesarean/  2. vbac.mp.  3. (vagina$ adj3 (birth$ or born or  deliver$) adj7 ((after$ or follow$ or  previous$ or prior or history) adj3  (cesarean$ or caesarean$))).mp.  4. 1 or 2 or 3  5. exp "Trial of Labor"/  6. (trial of labor or trial of labour).mp.  7. tol.mp.  8. 5 or 6 or 7  9. exp Cesarean Section, Repeat/  10. ((repeat$ or multip$ or another or prior  or previous$) adj3 (cesarean$ or  caesarean$)).mp.  11. ((second or third) adj cesarean).mp.  12. 9 or 10 or 11  13. 4 or 8 or 12  14. mental disease/ or exp mood disorder/  or exp anxiety disorder/ or anxiety/ or  exp psychosis/ or exp psychotropic  agent/  15. (mental health or mental illness or  psychological health or depression or  anxiety or post traumatic stress or  Post-traumatic stress or bipolar or  Schizophrenia or psychoses or  Psychosis or psychotic or  antidepressant or antipsychotic or  anxiolytics or antimanic or  psychotropic).mp.  16. 14 or 15  17. 13 and 16  18. limit 17 to (English language and  yr=”1980 – 2008”)  19. (exp animals/ or nonhuman/) not  human/  20. 18 not 19  21. conference*.pt  22. 20 not 21 | 1. exp Vaginal Birth after Cesarean/  2. vbac.mp. [mp=title, original title, abstract,  name of substance word, subject heading word]  3. (vagina$ adj3 (birth$ or born or deliver$) adj7  ((after$ or follow$ or previous$ or prior or  history) adj3 (cesarean$ or caesarean$))).mp.  4. 1 or 3 or 2  5. exp "Trial of Labor"/  6. (trial of labor or trial of labour).mp.  7. tol.mp.  8. 6 or 7  9. exp pregnancy/ or exp pregnancy complications/  10. exp Infant, Newborn/  11. exp child/  12. exp Obstetrics/  13. exp Obstetric Operation/  14. 9 or 10 or 11 or 12 or 13  15. 8 and 14  16. 15 or 5  17. exp Cesarean Section, Repeat/  18. ((repeat$ or multip$ or another or prior or  previous$) adj3 (cesarean$ or caesarean$)).mp.  19. ((second or third) adj cesarean).mp.  20. 17 or 18 or 19  21. 4 or 16 or 20  22. childhood obesity/  23. ((child* or pediatric or paediatric) adj3 obes*).mp.  24. exp neoplasm/  25. (leukaemia or leukemia or lymphoma or sarcoma  or Osteosarcoma or Neuroblastoma or  Retinoblastoma or Rhabdomyosarcoma or  cancer* or tumor* or tumour*).mp.  26. exp Infection/ or exp respiratory tract infection/ or  exp pneumonia/ or exp Influenza/ or exp  Bronchitis/ or exp diarrhea/ or exp gastroenteritis/  or exp meningitis/ or exp bacterial infection/  27. (infection or infectious or respiratory or pneumonia  or influenza or bronchitis or bronchiolitis or  gastroenteritis or diarrhea or diarrhoea or  meningitis or viral or bacterial).mp.  28. exp insulin dependent diabetes mellitus/  29. (type 1 diabetes or type i diabetes).mp.  30. asthma/  31. asthma*.mp.  32. exp inflammatory bowel disease/  33. (inflammatory bowel disease or crohn*disease or  colitis).mp.  34. 24 or 25 or 26 or 27 or 28 or 29 or 30 or 31 or 32  or 33  35. 21 and 34  36. limit 35 to (infant <to one year> or child  <unspecified age> or preschool child <1 to 6  years> or school child <7 to 12 years> or  adolescent <13 to 17 years>)  37. 21 and (22 or 23)  38. 36 or 37  39. limit 38 to (english language and yr="1980 –  2008")  40. conference*.pt.  41. 39 not 40 |
| --- | --- | --- |

**- EMBASE:**

| 1. vbac.mp.  2. (vagina$ adj3 (birth$ or born or deliver$)  adj7 ((after$ or follow$ or previous$ or  prior or history) adj3 (cesarean$ or  caesarean$))).mp.  3. 1 or 2  4. (trial of labor or trial of labour).mp.  5. tol.mp.  6. 4 or 5  7. ((repeat$ or multip$ or another or prior  or previous$) adj3 (cesarean$ or  caesarean$)).mp.  8. ((second or third) adj cesarean).mp.  9. 7 or 8  10. 3 or 6 or 9  11. mental disorders/ or Affective  Disorders/ or exp Major Depression/ or  exp "depression (emotion)"/ or exp  Anxiety Disorders/ or anxiety/ or exp  Bipolar Disorder/ or exp psychosis/ or  exp Antidepressant Drugs/  12. (mental health or mental illness or  psychological health or depression or  anxiety or post traumatic stress or  Post-traumatic stress or bipolar or  Schizophrenia or psychoses or  Psychosis or psychotic or  antidepressant or antipsychotic or  anxiolytics or antimanic or  psychotropic).mp.  13. 11 or 12  14. 10 and 13  15. limit 14 to (english language and yr="1980 -Current")  16. exp animals/ or animal models/  17. 15 not 16 |
| --- |

**- PsycINFO:**

**9,788** articles identified by search strategies

**3,948** duplicates

**5,840** articles screened on title and abstract

**5,597** articles excluded

**243** articles screened on full text

**196** articles excluded

**47** studies included in review^1^

**Figure S1. Flow diagram of study selection process**

^1^ Literature from October 2009-March 2022 for all outcomes apart from women’s mental health and health problems in childhood, where literature from 1980-March 2022

**Table S1.** **Characteristics of the studies included in the structured review**

| **First author, Year published** | **Study design** | **Location** | **Total number** | | **Recruitment period** | **Population** | **Maternal Outcomes** | **Baby/child outcomes** | **Considered/adjusted for potential confounding factors** |
| --- | --- | --- | --- | --- | --- | --- | --- | --- | --- |
|  |  |  | **Planned VBAC** | **ERCS** |  |  |  |  |  |
| Al-Zirqi, 2010^(34)^ | Population-based cohort | Norway | 11,954 (9,239 spontaneous & 2,715 induced labour onset) | 5,442 | 1999-2005 | Women with previous CS (number unknown but national guidelines state VBAC should only be attempted if 1 prior CS), who gave birth at ≥28 weeks’ gestation, excluding antepartum stillbirths, identified using a national birth registry | Uterine rupture | None | Adjusted for maternal age, ethnic origin, parity & gestational age |
| Cahill, 2010^(35)^ | Multicentre cohort | USA | 13,706 (12,535 after 1 prior CS; 1,082 after 2 prior CSs; 89 after ≥3 prior CSs) | 771  (all after ≥3 prior CSs) | 1996-2000 | Women with ≥ 1 prior CS, identified from 17 tertiary & community birth centres | Uterine rupture, blood transfusion, infection, surgical injury | None | No |
| Dekker, 2010^(36)^ | Population-based cohort | Australia | 10,958  (8,221 spontaneous onset of labour, no augmentation; 628 spontaneous onset of labour, augmentation with oxytocin; 2,109 labour induced) | 18,050 | 1998-2000 | Women with 1 prior CS only, who gave birth to a singleton infant, where the first birth was a live singleton, identified from the perinatal data collections of 4 states | Uterine rupture | None | Some relative effect measures adjusted for placental abruption |
| Law, 2010^(32)^ | RCT | Hong Kong | 145 | 146 | Not reported | Women with 1 prior lower segment CS and no previous vaginal births, who had a singleton pregnancy and were considered eligible for VBAC, recruited from a single University-affiliated tertiary referral unit | Mental health | None | Reported that there were no significant differences in baseline sociodemographic characteristics and psychometric scores between women randomised to planned VBAC and those randomised to ERCS |
| Cieminski, 2011^(37)^ | Single centre cohort | Poland | 492 (all spontaneous labour onset) | 228 | 1992-2002 | Women with prior CS (number not stated) via low transverse incision, who gave birth at a single hospital | Uterine rupture | None | No |
| Homer, 2011^(38)^ | Population-based cohort | Australia | 21,832 | 31,622 | 1998-2006 | Women with 1 prior CS, who gave birth to a vertex presenting singleton infant at ≥ 37 weeks’ gestation, identified from the perinatal data collection of one state |  | Neonatal intensive care unit admission | No |
| Crowther, 2012^(33)^ | Multicentre patient preference cohort with nested RCT | Australia | 1,237  (1,225 patient preference & 12 randomised) | 1,108  (1,098 patient preference & 10 randomised) | 2002-2007 | Women with 1 prior CS, presenting with a live cephalic presenting singleton fetus at ≥37 weeks’ gestation not including those with a prior vertical, inverted T or unknown uterine incision, previous uterine rupture, previous uterine surgery, previous uterine perforation, any contraindication to VB, cephalo-pelvic disproportion, lethal congenial anomaly or fetal anomaly associated with mechanical difficulties at birth, recruited from 14 maternity hospitals | Mortality, Uterine rupture, hysterectomy, haemorrhage, infection, surgical injury, length of hospital stay, pelvic floor dysfunction/perineal trauma | Perinatal mortality, neonatal mortality, respiratory intervention/ morbidity, hypoxic-ischaemic encephalopathy/ asphyxia, neonatal sepsis, birth trauma, neonatal intensive care unit admission, low Apgar score | Adjusted for key prognostic variables where there was an imbalance between the comparison groups: maternal socioeconomic status, BMI & indication for previous CS |
| Fitzpatrick, 2012^(78)^ | Population-based case-control | UK | Estimated number: 56,246 | Estimated number: 71,585 | 2009-2010 | All women identified as having a uterine rupture using a prospective national surveillance system covering all obstetrician-led maternity units in country, & a control group of women who gave birth to a fetus or infant who had not had a uterine rupture & who had given birth by CS in any previous pregnancy, identified from random sample of obstetrician-led maternity units in country in month 4 & 12 of study, weighted by total number of births. | Uterine rupture | None | Adjusted for maternal age, ethnicity, BMI, parity, number of previous CSs, other previous uterine surgery, time since last CS & last menstrual period, placenta praevia & macrosomia |
| Fruscalzo, 2012^(39)^ | Single centre cohort | Italy | 170 | 24 | 2007-2008 | Women with 1 prior CS not including those with a prior T incision or previous full thickness uterotomy, metroplasty or myomectomy, presenting with a singleton pregnancy with the foetus in cephalic presentation or twin pregnancy with the foetuses in cephalic-cephalic presentation, identified at a single hospital | Uterine rupture, haemorrhage, blood transfusion, infection | Neonatal intensive care unit admission | No |
| Gilbert, 2012^(40)^ | Secondary analysis of multicentre cohort | USA | 3,981  (all spontaneous labour onset) | 3,981 | 1999-2002 | Women with 1 prior CS eligible to plan VBAC, who gave birth to a live, vertex presenting singleton infant without aneuploidy or congenital malformations at ≥ 37 weeks’ gestation by ERCS (without indication other than prior CS) or planned VBAC where labour began spontaneously, identified from 19 centres of the Eunice Kennedy Shriver National Institute of Child Health & Human Development Maternal–Fetal Medicine Units Network | Mortality, uterine rupture, hysterectomy, infection, surgical injury | Neonatal mortality, respiratory intervention/ morbidity, hypoxic-ischaemic encephalopathy/ asphyxia, neonatal sepsis | Propensity score method used to identify ERCS & planned VBAC groups with comparable characteristics (including race, marital status, antenatal care, private medical payment, BMI, maternal age, prior CS indication, interval since last CS, whether had prior VB or prior VBAC, EDD confirmed by 1st trimester ultrasound, diabetes, asthma, thyroid disease, seizure disorder, chronic hypertension, heart disease, cigarette use, alcohol use, street drug use, preterm labour requiring tocolysis, infection, antepartum antibiotic administration, gestational hypertension/ preeclampsia, other maternal comorbidity & recruitment centre) |
| Holm, 2012^(41)^ | Population-based cohort | Denmark | 14,173 | 8,955 | 2001-2008 | Births at all hospitals using local electronic transfusion databases at the time of birth, identified using a national birth registry linked to a national transfusion database | Blood transfusion | None | No |
| Wen, 2012^(42)^ | Population-based cohort | USA | 557,772 | 1,275,635 | 1995-2002 | Women with 1 prior CS, who gave birth to a live singleton infant in their second pregnancy, identified using national linked live birth-infant death data | None | Neonatal mortality, respiratory intervention/ morbidity, birth trauma, low Apgar score | Adjusted for maternal age, race, marital status, education, cigarette smoking during pregnancy, first trimester visit, & infant sex |
| Fagerberg, 2013^(43)^ | Population-based cohort | Sweden | 41,450 | 18,193 | 1992-2007 | Women having their second birth, excluding multiple pregnancies, identified using a national birth registry | None | Low Apgar score | Some relative effect measures adjusted for year of birth, maternal characteristics, preterm birth, breech presentation, & birth weight standard deviation scores |
| Melamed, 2013^(44)^ | Single centre cohort | Israel | 93 | 111 | 1996-2011 | Women with 1 prior CS at ≥37 weeks’ gestation following a failed trial of operative VB, who gave birth to a subsequent singleton infant at ≥37 weeks’ gestation, excluding those with a prior vertical uterine incision & those who needed an urgent CS prior to labour onset in the subsequent pregnancy because of maternal &/or neonatal complications or major fetal anomalies, identified at a single university-affiliated tertiary medical centre | Uterine rupture, haemorrhage, surgical injury, birth trauma , length of hospital stay | Neonatal intensive care unit admission, low Apgar score | No |
| Regan, 2013^(45)^ | Population-based cohort | USA | 5,614 | 25,897 | 2006-2007 | Women with ≥ 1 prior CS who gave birth to a live singleton infant at 20-42 weeks’ gestation, excluding births complicated by major congenital malformations, identified in one state | Breastfeeding | None | Adjusted for maternal age, gestational hypertension, whether had no prenatal care, BMI, maternal race, & whether Medicaid recipients |
| Schmitz, 2013^(46)^ | Single centre cohort | France | 3,544 (1,828 spontaneous labour; 1,716 induction or augmentation of labour) | 593 | 1988-2010 | Women with 1 prior CS not including those with a prior classical uterine incision, who gave birth to a cephalic presenting singleton infant at ≥ 37 weeks’ gestation, identified at a single University hospital | Mortality, uterine rupture, haemorrhage, surgical injury, | Respiratory intervention/ morbidity, hypoxic-ischaemic encephalopathy/ asphyxia, neonatal intensive care unit admission, low Apgar score | No |
| Shatz, 2013^(47)^ | Single centre cohort | Israel | 5,839 (4,263 spontaneous labour; 1,576 induced labour) | 1,916 | 1988-2005 | Women with 1 prior CS via low transverse incision, who gave birth to a singleton vertex presenting infant without chromosomal abnormalities or structural defects, excluding those with contraindications to VBAC (including previous classical or inverted T uterine incision, placenta praevia, placenta accreta, placental abruption, antepartum death, severe preeclampsia, & non-reassuring fetal heart rate monitoring at presentation), identified at a single tertiary University Medical centre | Mortality, uterine rupture, hysterectomy, haemorrhage, blood transfusion, infection, | Perinatal mortality, neonatal mortality, hypoxic-ischaemic encephalopathy/ asphyxia, birth trauma, low Apgar score | No |
| Studsgaard, 2013^(48)^ | Single centre cohort | Denmark | 1,161 | 622 | 2003-2010 | Women with 1 prior CS & no diabetes, who gave birth to a singleton infant at ≥37 weeks’ gestation by planned VBAC or ERCS without contraindications for VBAC, not including antepartum stillbirths, identified at a single University hospital | Uterine rupture, haemorrhage | Hypoxic-ischaemic encephalopathy/ asphyxia, neonatal intensive care unit admission, low Apgar score | Some relative effect measures adjusted for prior vaginal deliveries, age of mother, pre-pregnancy BMI, birth weight |
| Sananès, 2014^(49)^ | Multicentre cohort | France | 1,269  (1,045 spontaneous onset of labour; 224 labour induced) | 806 | 2007-2012 | Women with 1 prior CS, who gave birth to a singleton infant at a level 2 & a level 3 maternity hospital | Uterine rupture | Hypoxic-ischaemic encephalopathy/ asphyxia | No |
| Bickford, 2015^(50)^ | Population-based cohort | Canada | 11,340 | 22,472 | 2000-2008 | Women with 1 or 2 prior CSs, who gave birth to a singleton cephalic presenting infant at 37-41 weeks’ gestation, excluding those with gestational hypertension, pre-existing diabetes & cardiac disease, identified using the perinatal data registry of 1 province | Mortality, uterine rupture, hysterectomy, haemorrhage, blood transfusion, infection | Perinatal mortality, neonatal mortality, respiratory intervention/ morbidity, hypoxic-ischaemic encephalopathy/ asphyxia, birth trauma, neonatal intensive care unit admission, low Apgar score | Reported that sequential removal from full multivariate model of the following covariates did not alter relative risks by more than 10% so reported relative risks are unadjusted: maternal age, BMI, number of previous CSs, augmentation of labour, induction of labour, hospital size, whether single parent & birth weight |
| Kok, 2015^(51)^ | Population-based cohort | Netherlands | 4,109 (all with spontaneous onset of labour) | 4,109 | 2000-2007 | Women with 1 prior CS at ≥37 weeks’ gestation, having a second birth of a live singleton cephalic presenting infant without congenital abnormalities by ERCS at ≥39 weeks’ gestation or spontaneous planned VBAC at ≥37 weeks’ gestation, identified using a national perinatal registry | Uterine rupture, haemorrhage, blood transfusion | Neonatal mortality, respiratory intervention/ morbidity, neonatal sepsis, birth trauma, low Apgar score | Propensity score method used to identify ERCS & planned VBAC groups with comparable characteristics (including race, socio-economic status; 1st pregnancy history: IVF, maternal age, non-gestational diabetes, pre-existing hypertension, pregnancy induced hypertensive disorder, spontaneous labour, malpresentation, ECS, gestational age, birth weight, Apgar score at 5 min, male gender, haemorrhage, transfusion; 2nd pregnancy: pregnancy interval <15 months, IVF, maternal age, gestational age) |
| Miller, 2015^(52)^ | Secondary analysis of multicentre cohort | USA | 4,252  (all induction of labour; 4,100 had 1 prior CS; 152 had 2 prior CSs) | 6,010 (all after 2 prior CSs) | 1999-2002 | Women with 1-2 prior CSs via low transverse or unknown incision & no contraindication to VB, who gave birth to a singleton infant with no anomaly, excluding antenatal stillbirths, identified from 19 centres of Eunice Kennedy Shriver National Institute of Child Health & Human Development Maternal–Fetal Medicine Units Network | Uterine rupture, hysterectomy, blood transfusion, infection, surgical injury | Hypoxic-ischaemic encephalopathy/ asphyxia, neonatal intensive care unit admission, low Apgar score | No |
| Nair, 2015^(53)^ | Population-based nested cohort | UK | Peripartum hysterectomy study - estimated number: 48,036; Peripartum haemorrhage study - estimated number: 67,269; Sepsis study - estimated number: 45,861 | Peripartum hysterectomy study - estimated number: 61,136; Peripartum haemorrhage study - estimated number: 85,616; Sepsis study - estimated number: 58,370 | Peripartum hysterectomy study: 2005-2006 Peripartum haemorrhage study: 2007-2009 Sepsis study: 2011-2012 | All women who had a peripartum hysterectomy, severe sepsis, major obstetric haemorrhage resulting in massive transfusion or failed tracheal intubation, excluding those without a prior history of CS & those with placenta praevia/accreta/percreta diagnosed before birth, identified in 4 previous national studies conducted at different time periods using a prospective national surveillance system covering all obstetrician-led maternity units in country. Control women also available for peripartum hysterectomy, severe sepsis, & failed tracheal intubation studies (no further details given) | Hysterectomy, haemorrhage, infection |  | Some effect estimates adjusted for current & previous pregnancy problems, number of previous CSs, pre-existing medical problems, parity, smoking status, socioeconomic status, ethnic background, marital status, BMI & maternal age |
| Black, 2016 (^54^) | Population-based cohort | Scotland | 4,250-22,226 depending on outcome | 3,388-17,919 depending on outcome | 1993-2007 | Second-born singleton term births to women with 1 previous CS, excluding stillbirths, identified from linked routinely-collected data | None | Neurodevelopment, health problems in childhood | Adjusted for maternal age, gestation at birth, area deprivation measure, maternal smoking status, birthweight, year of delivery, infant gender, breastfeeding at 6 weeks. Some effect estimates additional adjusted for maternal BMI, maternal salbutamol prescription, &/or maternal type 1 diabetes |
| Stattmiller, 2016^(55)^ | Population-based cohort | USA | 144, 066 | 541, 071 | 2003-2011 | Women with prior CS (number not stated), excluding those with indication for ERCS (including diabetes, hypertension, preterm, soft-tissue disorders, malpresentation, mental health disorders, asthma, thyroid abnormalities, isoimmunisation, antenatal bleed, oligohydramnios, herpes, multiple pregnancy, intra-uterine growth restriction, polyhydramnios, cardiovascular diseases, stillbirth, unengaged fetal head, substance use, uterine scar not from previous CS, renal abnormalities, congenital fetal anomalies, systemic lupus erythematosus, liver abnormalities, cerebral haemorrhage), identified using database of nationwide sample of hospital inpatient stays | Mortality, uterine rupture, hysterectomy, haemorrhage, blood transfusion, infection | None | Adjusted for maternal age, race, median household income, hospital, region, primary payer & year |
| Vandenberghe, 2016^(56)^ | Population-based cohort | Belgium | 12,754 | 14,253 | 2012-2013 | All women identified as having a uterine rupture using a prospective national surveillance system covering 97% of maternity units in the country. Data on denominators available from the national perinatal registry covering nearly all births in maternity units & at home in the country | Uterine rupture | None | No |
| Vigorito, 2016^(57)^ | Single centre cohort | Italy | 10  (all without induction of labour or oxytocin) | 40 | 2011-2015 | Women with 3 prior CSs & ≥ 1 prior VB, who gave birth to a singleton vertex presenting infant at term not including those who had a CS for medical reasons (e.g. placenta praevia, non-reassuring patterns in the intrapartum or antepartum fetal heart monitoring, medical condition precluding a planned VBAC), referred to a single centre | Uterine rupture, hysterectomy, blood transfusion | Neonatal intensive care unit admission | No |
| Colmorn, 2017^(58)^ | Multicountry population-based cohort | Denmark, Finland, Norway & Sweden | 24,366 | 11,084 | 2009-2012 | Women having their second birth who had complete uterine rupture, abnormally invasive placenta, peripartum hysterectomy, or severe blood loss, identified from prospective surveillance system covering 91% of all Nordic births cross-checked with national medical birth registers, hospital discharge registers & transfusion databases. Data on denominators collected from national medical birth registers | Uterine rupture, hysterectomy, blood transfusion | None | Adjusted for maternal age, BMI & multiple pregnancy at the second pregnancy |
| Litwin, 2017^(59)^ | Population-based cohort | USA | 181,566 | 854,988 | 2011-2013 | Women with ≥ 1 prior CS, who gave birth to a live singleton cephalic presenting infant without congenital malformations at 37-40 weeks’ gestation, identified using national linked live birth-infant death data | None | Neonatal mortality, respiratory intervention/ morbidity, birth trauma, neonatal intensive care unit admission, low Apgar score | Adjusted for maternal age, race, education, marital status, smoking, birth weight, characteristic of labour (induced, augmented or artificial rupture of membrane) & comorbidities (gestational diabetes, pre-pregnancy hypertension, gestational hypertension) |
| O'Neill, 2017^(60)^ | Population-based cohort | Denmark | 40,685 | 20,941 | 1982-2010 | Women with 1 prior CS, having a second birth of a singleton cephalic presenting infant by planned VBAC or ERCS at 37-42 weeks’ gestation, excluding deaths due to congenital anomalies, identified using national routinely available data |  | Neonatal mortality | Adjusted for maternal age, maternal country of origin, educational attainment, mother & father's gross income, marital status, infant birthplace, infant birth weight, history of pregnancy loss, & birth year |
| Tsai, 2017^(61)^ | Single centre cohort | Taiwan | 73 | 204 | 2006-2015 | Women with 1 prior CS not including those with a classical or inverted-T incision, history of myomectomy, major brain lesions, major cardiac diseases, & severe pelvic trauma history, who gave birth at ≥37 weeks’ gestation by planned VBAC or ERCS, identified at a single hospital | Uterine rupture, haemorrhage | None | No |
| Gobillot, 2018^(62)^ | Single centre cohort | France | 248 (all induction of labour with oxytocin) | 165 | 2013-2017 | Women with 1 prior CS, who gave birth to a singleton infant after 37 weeks’ gestation, who did not go spontaneously into labour & did not have contraindications to planned VBAC (including previous uterine body incision, placenta praevia or abnormal pelvimetry with a breech presentation), identified at a single hospital | Uterine rupture, hysterectomy, haemorrhage, blood transfusion, infection, surgical injury, length of hospital stay | Hypoxic-ischaemic encephalopathy/ asphyxia, neonatal intensive care unit admission, low Apgar score | No |
| Gundersen, 2018^(63)^ | Population-based cohort | Denmark | 30,734 | 21,741 | 2004-2010 | All live births, identified using a national birth registry | Infection | None | Adjusted for age at delivery, smoking, BMI, educational level, gestational diabetes mellitus, infection during pregnancy, low birth weight, preterm birth, preterm prelabour rupture of membranes, pre-eclampsia & parity |
| Young, 2018^(64)^ | Population-based cohort | Canada | 58,704 | 138,836 | 2003-2015 | Women with 1 prior CS only, who gave birth to a singleton infant at 37-43 weeks’ gestation, identified from a database covering ~98% of all deliveries in the country | Uterine rupture, hysterectomy, haemorrhage, blood transfusion | Neonatal mortality, respiratory intervention/ morbidity | Adjusted for maternal age, diabetes mellitus, hypertension & labour induction |
| Fitzpatrick, 2019^(65)^ | Population-based cohort | Scotland | 28,464 (23,119 labour not induced; 5,245 labour induced) | 45,579 | 2002-2015 | Singleton births at 37-41 weeks’ gestation to women with ≥ 1 prior CS, excluding antepartum stillbirths & women with contraindications to planned VBAC (including non-cephalic presentation at birth, placenta previa, abdominal pregnancy, known or suspected disproportion of maternal &/or fetal origin, tumour of corpus uteri, or birth by pre-labour non-elective caesarean section), identified from linked routinely-collected data | Uterine rupture, hysterectomy, blood transfusion, infection, surgical injury, length of hospital stay, breastfeeding | Perinatal mortality, respiratory intervention/ morbidity, neonatal intensive care unit admission, low Apgar score | Adjusted for year of birth, maternal age, mother’s country of birth, marital status, socioeconomic status, number of previous caesarean sections, any prior vaginal birth, interpregnancy interval, maternal smoking status at booking, maternal BMI at booking. Some effect estimates additional adjusted for hypertensive disorder, diabetes, prelabour rupture of membranes, sex of infant, gestational age at delivery, &/or birth weight centile |
| Huisman, 2019^(66)^ | Multicentre cohort | Netherlands | 993 (all had labour induced with a balloon catheter) | 321 | 2011-2012 | Women with 1 prior CS, who gave birth to a live singleton infant in cephalic presentation at 37-41 weeks’ gestation & had an unfavourable cervix & an indication for delivery, excluding contraindications for VB such as placenta previa, previous classical uterine incision or obstructing cervical fibroids, identified from 51 hospitals | Mortality, uterine rupture, haemorrhage, infection, length of hospital stay | Hypoxic-ischaemic encephalopathy/ asphyxia, neonatal intensive care unit admission, low Apgar score | Adjusted for maternal age, BMI, prior vaginal birth, unplanned previous caesarean section, reasons for labour induction & prolonged rupture of membranes |
| Lehmann, 2019^(67)^ | Population-based cohort | Norway | 30,176 (15,765 classified as “low-risk” & 14,411 classified as “high-risk” women) | 12,249  (4,658 classified as “low-risk” & 7,591 classified as “high-risk” women) | 1989-2009 | Singleton cephalic second births at ≥37 weeks’ gestation to women with 1 prior CS, identified using a national birth registry | None | Perinatal mortality, neonatal intensive care unit admission, low Apgar score | Adjusted for year of delivery, maternal age, education, country of origin, geographical region, & size of maternity unit |
| Modzelewski, 2019^(68)^ | Single centre cohort | Poland | 35 | 377 | 2010-2017 | Women with 2 prior CS, who gave birth to a singleton infant at term, excluding those with placenta previa, identified from a single tertiary hospital | Mortality, uterine rupture, hysterectomy, haemorrhage, blood transfusion, surgical injury | Neonatal death, hypoxic-ischaemic encephalopathy/ asphyxia, neonatal sepsis, low Apgar score | No |
| Pont, 2019^(69)^ | Population-based cohort | Australia | 25,497 | 60,471 | 2000-2012 | Women with 1 prior CS, who had a subsequent singleton birth at ≥37 weeks’ gestation, excluding those with contraindications to VBAC (including non-cephalic presentation, grand multipara, fetal anomalies recorded during pregnancy requiring maternal care, placenta accreta or previa, women with any hospital admissions noting a blood disorder, women with pre-existing hypertension or diabetes, other chronic medical conditions, evidence of previous uterine surgery or uterine rupture or prior classical caesarean or birth weight ≥5 kg at the prior caesarean), identified using perinatal data collection & hospital discharge data in 1 state | Haemorrhage, blood transfusion | None | Adjusted for maternal age, country of birth, number of prior vaginal births, gestational diabetes, history of postpartum haemorrhage, other pregnancy complications, gestational age, small-for-gestational-age, large-for-gestational-age, socioeconomic indexes for areas, & hospital type |
| Yao, 2019^(70)^ | Population-based cohort | USA | 81,257 | 457,007 | 2011-2014 | Obese (BMI≥30kg/m^2^) women with 1 or 2 prior CSs, who gave birth to a singleton infant without congenital anomalies at 37-42 weeks’ gestation, identified from a database capturing all births in the country | Uterine rupture, hysterectomy, blood transfusion | Neonatal death, respiratory intervention/ morbidity, neonatal intensive care unit admission, low Apgar score | Adjusted for maternal age, diabetes & hypertension |
| Dombrowski, 2020^(71)^ | Population-based cohort | USA | 847 | 25,030 | 2010-2012 | Women with 2 prior CSs, who gave birth to a live singleton infant without congenital anomalies at 37-42 weeks’ gestation, excluding those with clear contraindications to planned VBAC (including malpresentation, genital herpes, placenta previa, vasa previa), identified from linked hospital discharge & birth certificate data in 1 state | Length of hospital stay | Neonatal intensive care unit admission | Propensity score matching approach used to identify ERCS & planned VBAC groups with comparable characteristics |
| Rotem, 2020^(72)^ | Single centre cohort | Israel | 485 | 2,234 | 2005-2019 | Women with 2 prior CSs, giving birth between 24-42 weeks’ gestation, excluding those with contraindications to VB (including non-vertex presentation, placenta previa, placenta accreta, mullerian uterine anomalies, multifetal gestation other than twins, & women without at least 1 previous VB), identified from a single tertiary hospital | Uterine rupture, haemorrhage, blood transfusion, length of hospital stay | Respiratory intervention/ morbidity, hypoxic-ischaemic encephalopathy/ asphyxia, neonatal intensive care unit admission, | No |
| Takeya, 2020^(73)^ | Population-based cohort | Japan | 1,730 | 32,739 | 2013-2015 | Women with 1 prior CS, who gave birth to a singleton at 37-41 weeks’ gestation, excluding those with placenta previa, intrauterine fetal death or non-cephalic presentation, identified from a nationwide institution-based registry | Mortality, uterine rupture, hysterectomy, haemorrhage | Low Apgar score | No |
| Uno, 2020^(74)^ | Single centre cohort | Japan | 471 | 264 | 2005-2017 | Women with 1 prior CS, who gave birth to a singleton without lethal congenital anomalies at ≥34 weeks’ gestation, excluding those who had an antepartum stillbirth or contraindications to VB (including former caesarean not low transverse, obstetric contraindications, non-vertex presentation, history of myomectomy, CS for any condition that potentially threatened maternal life or incited non-reassuring fetal status before labour onset), identified from a single hospital | Uterine rupture | Hypoxic-ischaemic encephalopathy/ asphyxia, low Apgar score | No |
| Fitzpatrick, 2021^(75)^ | Population-based cohort | Scotland | 10,220 (8,220 labour not induced; 1,960 labour induced) | 20,911 | 2010-2015 | Women with ≥ 1 prior CS who gave birth to a singleton infant at 37-41 weeks’ gestation, excluding antepartum stillbirths, women with contraindications to planned VBAC (including non-cephalic presentation at birth, placenta previa, abdominal pregnancy, known or suspected disproportion of maternal &/or fetal origin, tumour of corpus uteri, or birth by pre-labour non-elective caesarean section) and women dispensed psychotropic medication in the year before they gave birth, identified from linked routinely-collected data | Mental health | None | Adjusted for year of birth, maternal age, mother’s country of birth, marital status, socio-economic status, number of previous caesarean sections, any prior vaginal birth, inter-pregnancy interval, any prior stillbirth or neonatal death, maternal smoking status at booking, maternal BMI at booking, hypertensive disorder, diabetes, any breastfeeding at 6-8 weeks postpartum |
| Fitzpatrick, 2021^(76)^ | Population-based cohort | Scotland | 18,851 (15,488 labour not induced; 3,305 labour induced) | 26,041 | 2002-2011 | Live singleton births at 37-41 weeks’ gestation to women with ≥ 1 prior CS, excluding births to women with contraindications to planned VBAC (including non-cephalic presentation at birth, placenta previa, abdominal pregnancy, known or suspected disproportion of maternal &/or fetal origin, tumour of corpus uteri, or birth by pre-labour non-elective caesarean section) & children who died before the age of 4 years, identified from linked routinely-collected data | None | Neurodevelopment | Adjusted for year of birth, maternal age, mother’s country of birth, marital status, socio-economic status, child’s ethnicity, number of previous caesarean sections, any prior vaginal birth, inter-pregnancy interval, maternal smoking status at booking, maternal BMI at booking, hypertensive disorder, diabetes, prelabour rupture of membranes, sex of infant, gestational age at birth, birthweight centile, any breastfeeding at 6-8 weeks postpartum |
| Wagner, 2021^(77)^ | Population-based cohort | USA | 38,649 | 38,649 | 2014-2018 | Women with 2 prior CSs, who gave birth to a singleton, cephalic, non-anomalous liveborn infant at 37-41 weeks’ gestation, identified using national vital statistics linked birth and infant death data | Uterine rupture, hysterectomy, blood transfusion | Neonatal mortality, respiratory intervention/ morbidity, low Apgar score | Propensity score method used to identify ERCS & planned VBAC groups with comparable characteristics (considering following characteristics: maternal age, race/ethnicity, education, marital status, prenatal care, chronic hypertension, pregestational diabetes, gestational diabetes, year of birth) |

Abbreviations: BMI, body mass index; CS, caesarean section; ECS, elective caesarean section; EDD, estimated date of delivery; ERCS, elective repeat caesarean section; IVF, in vitro fertilisation; RCT, randomised control trial; VB, vaginal birth; VBAC, vaginal birth after previous caesarean
